# Intelligent Field‐Adaptive Electrohydrodynamic Printing for Curved Conformal Electronics

**DOI:** 10.1002/advs.78343

**Published:** 2026-10-18

**Authors:** Chang Liu, Yiwen Feng, Xu Chen, Kaiwen Chen, Ran Zhang, Xiaopeng Zhang, Liujia Suo, Xiangji Chen, Zefei Li, Fengshu Wang, Ruixuan Xu, Shuaiwu Liu, Shiwen Liang, Lingjie Kong, Dazhi Wang

**Affiliations:** ^1^ State Key Laboratory of High‐Performance Precision Manufacturing Dalian University of Technology Dalian China; ^2^ Liaoning Huanghai Laboratory Dalian University of Technology Dalian China; ^3^ Ningbo Institute of Dalian University of Technology Ningbo China; ^4^ State Key Laboratory of Structural Analysis Optimization and CAE Software for Industrial Equipment Dalian University of Technology Dalian China

**Keywords:** alternating electric field, artificial neural network, curved conformal electronics, electrohydrodynamic printing, insulating substrates

## Abstract

Curved conformal electronics are increasingly important for wearable devices, biosensing, and advanced communication systems, requiring high‐resolution and high‐precision manufacturing on complex surfaces. Electrohydrodynamic printing is a promising method for fabricating conformal electronics, but its application on curved insulating substrates is hindered by local electric‐field disturbances arising from residual charge accumulation and spatial variations, leading to poor dimensional control. To address this challenge, an intelligent field‐adaptive electrohydrodynamic printing framework is established, in which alternating‐electric‐field modulation reduces the effects of residual charge, the equivalent electrical gap quantitatively characterizes spatially varying electric‐field conditions, and machine learning‐based parameter regulation compensates for local electric‐field variations. Based on this framework, a machine‐learning and alternating‐electric‐field synergistic modulated electrohydrodynamic printing approach is developed to achieve stable printing and precise line‐width control on curved substrates. The approach rapidly optimizes parameters for target line widths, achieving average relative errors around 10% or lower at the initial printing position, and further reduces average relative line‐width errors by up to 7.24 percentage points through adaptive regulation during curved‐substrate printing. Diverse high‐precision patterns are printed, and the conformal frequency‐selective surface and antenna with excellent electromagnetic performance are fabricated, demonstrating the potential of this framework for advancing curved conformal electronics manufacturing.

## Graphical abstract

To mitigate residual‐charge accumulation and spatial electric‐field variations during electrohydrodynamic printing on curved insulating substrates, an intelligent field‐adaptive framework combines alternating‐electric‐field modulation, equivalent electrical gap characterization, and machine‐learning‐guided parameter inversion and pathwise regulation. It enables stable deposition, precise line‐width control, and high‐precision fabrication of functional structures for curved conformal electronics.

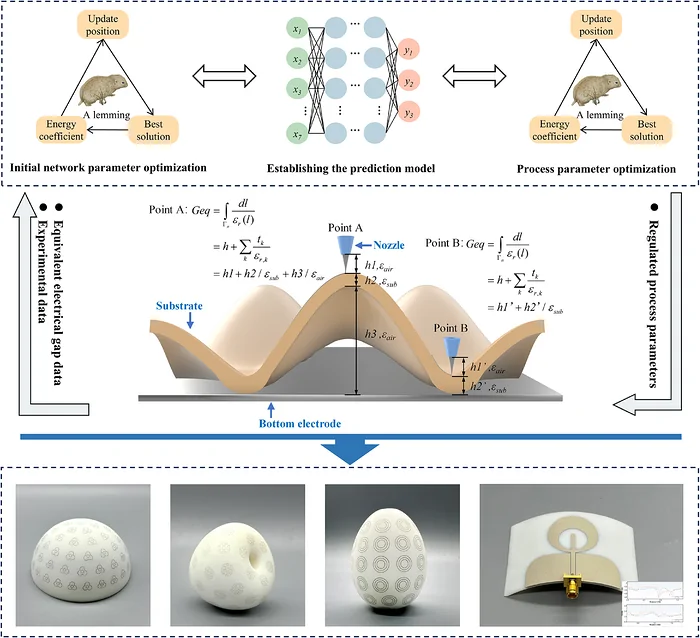

## 1 Introduction

Curved conformal electronics refers to electronic systems that can geometrically conform to non‐planar substrates, enabling functional integration on 3D surfaces [1]. With the escalating demand for 3D electronic systems and structure‐function integration, curved conformal electronics has garnered increasing attention and demonstrated extensive application potential in aeronautics and aerospace, biomaterials and medical technology, wearable devices, and advanced equipment [2, 3, 4]. Compared to the manufacturing of conventional planar electronics, the construction of curved conformal electronics is significantly restricted by complex spatial geometries. Currently, the main methods for manufacturing curved conformal electronics encompass curved surface lithography [5], transfer printing [6], inkjet printing [7], aerosol printing [8], and electrohydrodynamic (EHD) printing [9, 10]. Although curved surface lithography provides high resolution, it is restricted by the complexity of mask design and cost‐related factors. Transfer printing methods have limited applicability to non‐developable surfaces and those with complex convex‐concave features. Inkjet printing and aerosol jet printing offer advantages in process simplicity, fabrication flexibility, and damage‐free manufacturing. However, their dependence on droplet formation, atomization conditions, and ink physicochemical properties limits printing resolution and material adaptability, thereby restricting the dimensional accuracy and functional performance of fabricated structures. In contrast, EHD printing uses an electric field to generate fine liquid jets that are much smaller than the nozzle diameter, thereby enabling the deposition of functional microstructures with line widths smaller than the nozzle size [11]. As a result, EHD printing offers clear advantages in printing resolution, pattern precision, and material adaptability [12, 13]. Benefiting from these advantages, EHD printing has been increasingly explored for the fabrication of conformal electronics on curved surfaces [14, 15, 16].

However, the performance advancement of curved conformal electronics places increasingly stringent demands on fabrication accuracy and process stability. Since these devices are commonly constructed on curved insulating substrates, conventional EHD printing is constrained by local electric‐field fluctuations induced by residual charge accumulation and spatial variations during curved‐surface deposition, which greatly limits its further development for high‐precision conformal electronics manufacturing [17]. More specifically, the deposited liquid carries like charges during EHD printing, which are difficult to dissipate on insulating substrates [18]. As a result, residual charges accumulate on the substrate surface and disturb the local electric‐field distribution [19]. Meanwhile, during curved‐surface printing, the spatial relationship between the nozzle and the grounded electrode changes continuously along the printing path, further causing position‐dependent fluctuations in the driving electric field [20]. As the electric field governs both the printing state and structural dimensions, the combined effects of residual charge accumulation and position‐dependent electric field fluctuations directly lead to unstable printing and deposition‐size variations. This significantly increases the difficulty of dimensional control in conformal surface patterning, thereby hindering the reliable fabrication of delicate functional structures on complex curved surfaces [21, 22, 23]. Various strategies have been explored to mitigate local electric‐field variations arising from residual charge accumulation and spatial changes in nozzle position during EHD printing on curved insulating substrates. These include modifying the local electric field environment, such as introducing dielectric coatings [17] or removing ground electrodes while relying on self‐induced electric fields [24], as well as adjusting process parameters, including the applied voltage [20] or nozzle‐substrate distance [25]. However, these approaches exhibit significant limitations. The introduction of dielectric coating requires pre‐deposition of nanoparticle layers on the substrate surface, resulting in increased process complexity and limited adaptability to complex curved geometries. Methods relying on self‐induced electric fields can regulate the local electric‐field distribution to a certain extent and partially alleviate field fluctuations induced by variations in nozzle position. However, owing to the inherently insulating nature of the substrate, they have limited ability to suppress residual charge accumulation and the resulting electric‐field distortion. Moreover, they cannot fully compensate for the position‐dependent field fluctuations caused by continuous changes in the relative position between the nozzle and the grounded electrode. Parameter adjustment strategies can reduce the charge carried by the liquid but cannot fundamentally suppress the adverse effects of residual charges. Moreover, compensating for position‐induced electric field variations through empirical tuning of process parameters relies heavily on trial‐and‐error, leading to limited regulation accuracy, poor adaptability to complex curved surfaces, and substantial consumption of time and experimental resources. Overall, existing methods can only partially alleviate local electric‐field disturbances caused by residual charge accumulation and position‐dependent nozzle‐electrode variations during EHD printing on curved insulating substrates. These methods still exhibit clear limitations and cannot fundamentally resolve the field fluctuations that undermine printing stability and dimensional accuracy. More fundamentally, a unified field‐adaptive theoretical and technical system for this process has not yet been established. Such a system is required to mitigate the adverse effects associated with residual charge accumulation through an appropriate physical mechanism, quantitatively describe the influence of nozzle‐electrode positional variations on the local electric field, and compensate for position‐dependent field variations to maintain stable and high‐precision deposition.

To address local electric‐field disturbances arising from residual charge accumulation and position‐dependent effects during EHD printing, this work establishes a unified intelligent field‐adaptive theoretical and technical framework for high‐precision fabrication of conformal structures on curved insulating substrates. In this framework, alternating‐electric‐field modulation serves as the physical mechanism for mitigating residual‐charge effects, an equivalent electrical gap is introduced as a quantitative descriptor of position‐dependent electric field variations, and machine‐learning‐guided multi‐parameter regulation is used to compensate for these field variations and maintain stable, consistent line‐width formation.

In this paper, based on the intelligent field‐adaptive EHD printing theoretical and technical framework, a machine‐learning and alternating‐electric‐field synergistic modulated electrohydrodynamic printing approach, abbreviated as ML‐AE‐EHD printing, is developed. Within this approach, an alternating electric field is introduced to establish stable printing conditions through dynamic charge neutralization [26, 27], thereby enhancing electric field stability, and experimental data are collected to develop a predictive model that characterizes the mapping from process parameters and nozzle position to the resulting printing state and line‐width dimensions. An equivalent electrical gap is further introduced to represent position‐dependent electric field conditions as a unified model input. Optimal process parameters required to achieve target line widths under stable printing conditions are then determined through inverse optimization based on the intelligent optimization algorithm, enabling rapid and reliable identification of suitable parameter combinations without reliance on empirical trial‐and‐error, while establishing a robust parameter baseline for subsequent regulation. Intelligent and coordinated regulation of multiple process parameters is further implemented along the printing path on curved insulating substrates in response to variations in the equivalent electrical gap, enabling effective compensation for electric field fluctuations induced by spatial variations in nozzle position, thereby ensuring stable printing and consistent, high‐precision control of line‐width dimensions across complex curved surfaces. The proposed ML‐AE‐EHD printing approach achieves high‐precision patterning with controllable line widths on diverse insulating curved substrates and enables the fabrication of a curved conformal frequency‐selective surface (FSS) [28, 29, 30] and a curved conformal antenna [31, 32, 33] with excellent electromagnetic performance. Successful high‐precision and stable manufacturing on curved insulating substrates not only verifies the effectiveness of the ML‐AE‐EHD printing approach but also confirms the validity and practicality of the proposed intelligent field‐adaptive theoretical and technical framework. These results demonstrate that this work advances the manufacturing level of conformal electronics and provides technical support for their further development.

## 2 Materials

In this work, a signal generator (Keysight 33521B) provides electrical signals with defined voltage amplitude, frequency, and duty cycle. The signals are amplified by a high‐voltage amplifier (Trek 10/10B‐HS) and applied between the nozzle and the curved insulating substrate to generate a controllable electric field. A five‐axis motion platform governs the relative motion between the nozzle and the substrate, enabling precise control of the velocity and the nozzle‐substrate distance. The nozzle orientation is adjusted in real time so that its axis remains aligned with the normal local surface of the curved substrate. A dispensing machine (MUSASHI V2) supplies back pressure to ensure continuous and stable delivery of the paste during printing. The output of the high‐voltage amplifier is connected to the needle, which functions as the upper electrode, while a metal plate placed beneath the curved insulating substrate is grounded and serves as the bottom electrode. In this work, a nano‐silver paste (Shenzhen Sryeo Electronic Paste Co., Ltd.) with a solids content of 85% and silver particle sizes ranging from 300 to 500 nm is used as the printing material. A stainless‐steel nozzle with an outer diameter of 100 µm is used for the printing experiments. Alumina ceramic is selected as the printing substrate. Prior to printing, the substrate is ultrasonically cleaned in absolute ethanol for 10 min and subsequently dried under a nitrogen stream.

## 3 Methodology

Figure 1 shows the proposed intelligent field‐adaptive EHD printing theoretical and technical framework and its validation in stable, high‐precision printing on curved insulating substrates. Figure 1a illustrates how alternating‐electric‐field modulation suppresses residual charge accumulation and mitigates the associated electric‐field distortion within the intelligent field‐adaptive EHD printing theoretical and technical framework. Neutralization between positive and negative surface charges changes the surface‐charge distribution and consequently reconfigures the local electric field, thereby mitigating residual‐charge effect [34]. The printing process on curved insulating substrates involves dynamic charge compensation through the directional migration and redistribution of electrons and cations within the silver paste under the applied alternating electric field, thereby enhancing printing stability. The liquid bridge [35] is primarily governed by two driving forces: the electric field force induced by the alternating electric field and the mechanical pulling force exerted by the substrate [36]. The magnitude of the electric field force depends on the local electric field strength, which is governed by multiple coupled factors. These factors include the voltage applied between the nozzle and the ground electrode, the spatial distance between the nozzle and the ground electrode, the distance between the nozzle and the substrate, and the dielectric properties of the substrate [37, 38, 39]. Under the conditions of a curved insulating substrate, as the nozzle moves along the printing path on the curved surface, both the geometric distance between the nozzle and the ground electrode and the substrate structure vary with the spatial position [40]. This causes the electric field between the nozzle and the substrate to change with the spatial position, thereby affecting the stability of the liquid bridge and the line‐width dimensions of the deposition.

**FIGURE 1:**
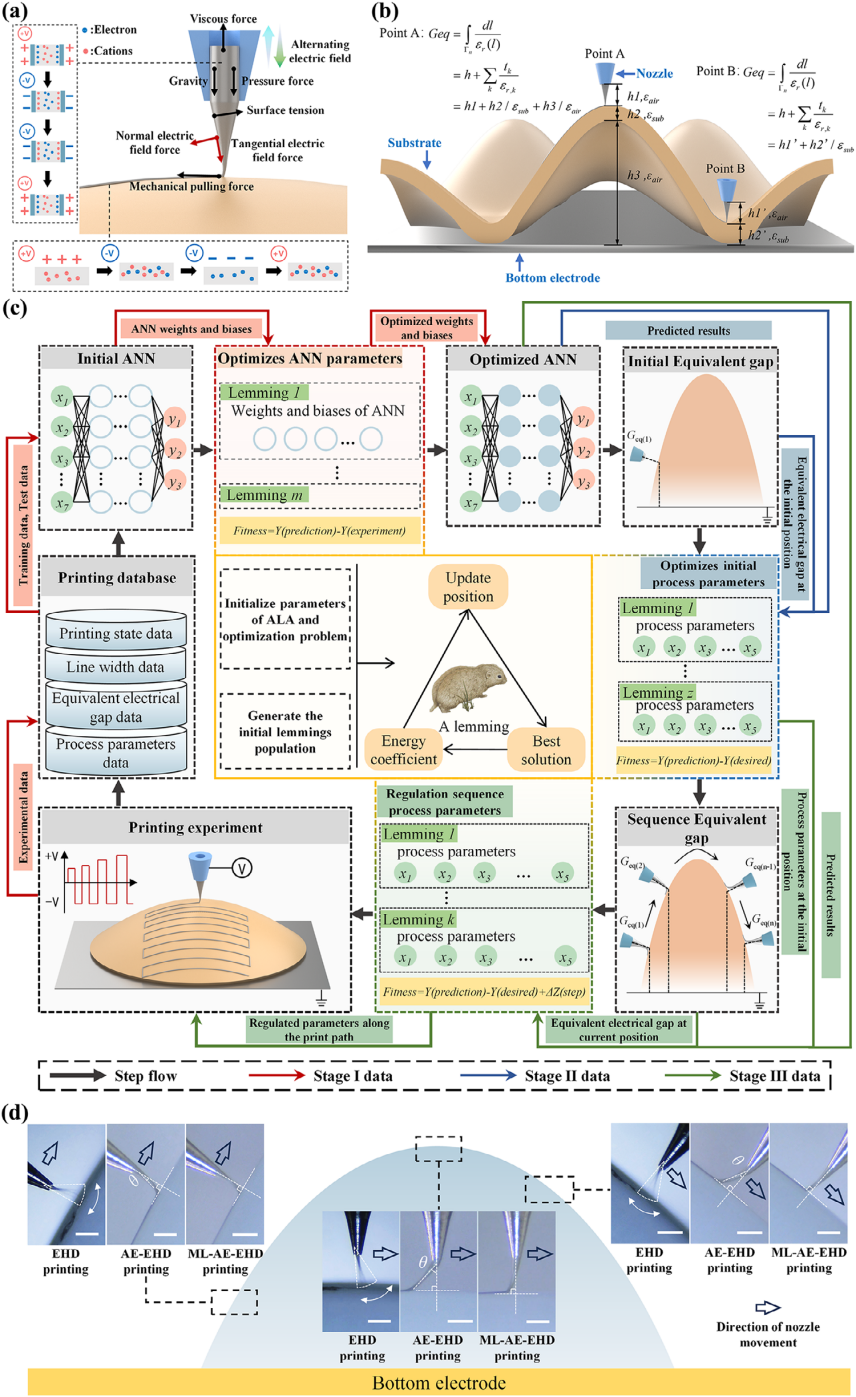
Intelligent field‐adaptive EHD printing theoretical and technical framework. (a) physical mechanism for suppressing residual charge accumulation; (b) schematic of the equivalent electrical gap; (c) machine‐learning‐guided multi‐parameter regulation; (d) the effect of ML‐AE‐EHD printing approach on the liquid bridge. Scale bars, 300 µm.

Within the intelligent field‐adaptive EHD printing theoretical and technical framework, equivalent electrical gap (*Geq*) is introduced as a quantitative descriptor of position‐dependent electric‐field conditions by integrating geometric distance and substrate dielectric properties. At the method level, *Geq* serves as a model input in ML‐AE‐EHD printing, allowing the local electric‐field condition at each nozzle position and its variation along the curved printing path to be incorporated into the predictive model.

Since the characteristic dimensions of the printing system are much smaller than the electromagnetic wavelength corresponding to the applied alternating voltage, the system can be treated under the quasi‐static approximation [41]. Under the quasi‐static approximation, Maxwell's equations yield an irrotational electric field, satisfying:

(1) $$\nabla \times E_{t}=0$$

where ***E*** *_t_* denotes the instantaneous electric field. Accordingly, the instantaneous voltage between the nozzle at potential *V_t_* and the grounded electrode at zero potential can be expressed as:

(2) $$V_{t}=\int_{\Gamma_{n}}E_{t}\cdot dl$$

where Γ*_n_* denotes the dominant electric field path between the nozzle and the ground electrode, and *d* ***l*** represents the differential arc length along this path. The electric displacement field and the electric‐field strength in a dielectric medium are related by:

(3) $$D_{t}=\varepsilon_{o}\varepsilon_{r}E_{t}$$

where ***D*** *_t_* is the instantaneous electric displacement field, *ε_o_* is the vacuum permittivity, and *ε_r_* is the relative permittivity of the medium. In the air‐dielectric configuration considered here, the dominant electric‐field path passes through media with different relative permittivities. Therefore, its geometric length alone cannot fully characterize the electrical separation between the nozzle and the grounded electrode. The media along this path are treated as linear and isotropic dielectrics in series, with the relative permittivity of each medium taken as constant under the experimental conditions. The voltage‐drop contribution of a path segment of length *dl* and relative permittivity *ε_r_* (*l*) is therefore proportional to *dl*/*ε_r_* (*l*). Accordingly, the equivalent electrical gap is defined as:

(4) $$G_{eq}=\int_{\Gamma_{n}}\frac{dl}{\varepsilon_{r}\left(l\right)}$$

where *l* is the arc‐length coordinate along Γ*_n_*, and *ε_r_* (*l*) is the local relative permittivity. Physically, *G_eq_* represents the effective electrical separation between the nozzle and the grounded electrode by incorporating both the length of the dominant electric‐field path and the dielectric properties of the intervening media. For the curved‐surface printing configuration considered here, the dominant electric‐field path consists of the nozzle‐to‐substrate air gap and one or more dielectric layers between the substrate surface and the grounded electrode. Accordingly, Equation (4) can be expressed as:

(5) $$G_{eq}=h+\sum_{k}\frac{t_{k}}{\varepsilon_{r,k}}$$

where *h* denotes the nozzle‐to‐substrate air gap, *t_k_* denotes the effective path length through the *k*th dielectric layer along the dominant electric‐field path, and *ε_r_,_k_* is the corresponding relative permittivity. During curved‐surface printing, the spatial configuration of the nozzle, curved substrate, and grounded electrode varies along the printing path. Consequently, the effective dielectric path lengths vary with the printing position, resulting in a position‐dependent *G_eq_* even when the nozzle‐to‐substrate air gap *h* is maintained constant. Under the assumptions stated above, *G_eq_* quantitatively combines path geometry and dielectric properties into a scalar descriptor of the electrical conditions at each printing position. At this level of description, fringing‐field effects are not treated as an independent contribution. As illustrated in Figure 1b, the *G_eq_* changes with the printing position on the curved substrate and serves to describe the local electric‐field condition at each position as well as its position‐dependent variation along the printing path.

As shown in Figure 1c, the ML‐AE‐EHD printing approach serves as the execution layer of the intelligent field adaptive EHD printing framework. It consists of three integrated stages: process modeling based on machine learning, determination of optimal process parameters for target line widths, and intelligent regulation of process parameters along printing paths on curved substrates. **In Stage I**, an alternating electric field is introduced to alleviate residual charge accumulation and establish stable printing conditions. Moreover, experimental data are collected to construct a predictive model based on an artificial neural network(ANN) [42]. To enhance model performance, the artificial lemming algorithm (ALA) [43, 44, 45] is employed to optimize network weights and biases, improving convergence behavior and generalization capability. An equivalent electrical gap is further introduced as a unified model input to represent the position‐dependent electric field conditions. This enables the model to capture the nonlinear coupling among process parameters, nozzle position, and the resulting printing state and line‐width dimensions. **In Stage II**, intelligent inverse optimization is performed to determine the process parameters that achieve the target line width at the initial position under stable printing conditions, where the discrepancy between the model‐predicted output and the target printing state and line‐width dimensions is defined as the fitness function of ALA (The fitness function is shown in Equation S2). Through iterative updates of individual positions, optimal parameter combinations are efficiently identified without reliance on empirical trial‐and‐error, establishing a robust parameter baseline for subsequent path‐dependent regulation. **In Stage III**, building upon this baseline, intelligent regulation of process parameters is implemented along the printing path on curved substrates. The equivalent electrical gap is first calculated at each discrete position along the printing path to characterize position‐dependent electric field conditions, and process parameters corresponding to each position are then determined through ALA‐based inverse optimization. The fitness function incorporates both the deviation between predicted and target printing outcomes and a smoothness constraint on parameter variation between adjacent positions, suppressing abrupt parameter changes and ensuring parameter continuity (The fitness function is shown in Equation S4). These position‐specific parameters are subsequently applied to regulate the printing process along the printing path, enabling coordinated adaptation to position‐dependent electric field conditions. As a result, stable and continuous printing is achieved while maintaining high‐precision control of line‐width dimensions across complex curved surfaces. These stages collectively constitute the ML‐AE‐EHD printing approach, enabling charge neutralization and intelligent parameter regulation for stable and precise electrohydrodynamic printing on curved insulating substrates.

Figure 1d illustrates the liquid bridge states under different printing approaches on curved insulating substrate. Conventional EHD printing uses a unidirectional direct‐current electric field to drive material deposition. AE‐EHD printing introduces alternating‐electric‐field modulation to mitigate residual‐charge‐related disturbances and improve liquid‐bridge stability. Building on AE‐EHD printing, ML‐AE‐EHD printing further incorporates the equivalent electrical gap and machine‐learning‐assisted coordinated regulation of multiple process parameters to compensate for position‐dependent electric‐field variations on curved substrates. In conventional EHD printing, residual charges have a tendency to accumulate on the curved insulating surface. This accumulation causes the liquid bridge to be disrupted by Coulomb forces [46], resulting in significant deflection and whipping, and ultimately reducing the printing stability.

By contrast, in AE‐EHD printing, the alternating electric field enables periodic charge neutralization, thereby effectively weakening the influence of residual charges on the liquid bridge. As a result, the whipping behavior of the liquid bridge is significantly suppressed, and the printing stability is improved. However, during printing on curved insulating substrates, as the nozzle moves along the printing path, the spatial relationship between the nozzle and the grounded electrode continuously changes. Accordingly, the equivalent electrical gap varies, which further causes changes in the electric field strength between the nozzle and the substrate. Without process parameter regulation, the liquid bridge tends to deflect in response to these local electric field variations, and this deflection becomes more pronounced with increasing equivalent electrical gap, thereby weakening the printing stability under curved‐surface conditions. In contrast, ML‐AE‐EHD printing compensates for the electric field fluctuations induced by changes in the equivalent electrical gap through the intelligent coordinated regulation of multiple process parameters, enabling the liquid bridge to maintain deposition along the local normal direction of the curved surface throughout the printing process. The results indicate that ML‐AE‐EHD printing not only reduces the influence of residual charges on liquid bridge stability by means of the alternating electric field, but also further suppresses the adverse effects of electric field disturbances caused by spatial position changes on liquid bridge behavior through intelligent process parameter regulation along the printing path, thereby effectively improving the stability, consistency, and controllability of deposition behavior on curved surfaces.

During the printing process, the printing state demonstrates multiple modes. A stable liquid bridge can only be formed when the printing state is favorable, which allows for the deposition of line widths with high consistency. Therefore, to precisely achieve the formation of the target line width on curved substrates, the constructed model must simultaneously predict both the printing state and line width dimensions, thus ensuring that the optimized parameters have practical feasibility [47, 48]. This work employs an ANN model to predict printing state and line width dimensions. To construct the ANN model within the ML‐AE‐EHD printing approach for curved insulating surfaces, seven key process variables that have a significant influence on the printing state and line‐width dimensions are carefully selected. These variables encompass: voltage amplitude (ranging from 1.0 to 3.1 kV), frequency (ranging from 10 to 800 Hz), duty cycle (ranging from 4 to 97%), equivalent electrical gap (ranging from 0.65 to 10.45 mm), nozzle‐substrate distance (ranging from 150 to 450 µm), velocity (ranging from 1 to 8 mm/s), and back pressure (ranging from 15 to 100 kPa). The dataset used in this work is collected from printing experiments conducted on semicylindrical substrates with curvature radii ranging from 5 to 100 mm. To ensure adequate coverage and sampling resolution within the 7D process‐parameter space while considering the experimental workload, Latin Hypercube Sampling (LHS) [49, 50, 51] is used to sample 1200 process‐parameter combinations. After normalization of each process parameter, the centered *L*_2_‐discrepancy of the 1200 sample points is 0.0081, while the mean and standard deviation of the nearest‐neighbor distances are 0.3184 and 0.0650, respectively. Moreover, 95.42% of the sample points have nearest‐neighbor distances within the range of the mean plus or minus two standard deviations (shown in Figure S1). These quantitative results indicate that the samples provide uniform and adequate coverage of the process‐parameter space, with good overall space‐filling characteristics, balanced local spacing, and no obvious clustering or spatial isolation. For each process‐parameter combination, the printing experiment is independently repeated three times to ensure the reliability of the experimental results. The resulting dataset is subsequently divided into training and test sets at a ratio of 75:25 for model training and generalization performance evaluation. All printing experiments used for dataset construction were conducted on substrates with semicylindrical curved surfaces. Accordingly, the training set consists entirely of curved‐substrate samples covering curvature radii ranging from 5 to 100 mm.

Figure 2a depicts the topological structure of the ANN model in Stage I of the ML‐AE‐EHD printing approach for curved surfaces. The model consists of an input layer, three hidden layers, and an output layer. The input layer is composed of seven neurons, with each neuron corresponding to one of the seven key process parameters. The output layer consists of three neurons, which respectively represent a non‐conforming printing state (Printing N), a favorable printing state (Printing Y), and the line width dimension. The detailed parameter settings of ANN are shown in Table S1. It should be noted that the line‐width output has practical engineering significance only under favorable‐printing conditions (Printing Y).

**FIGURE 2:**
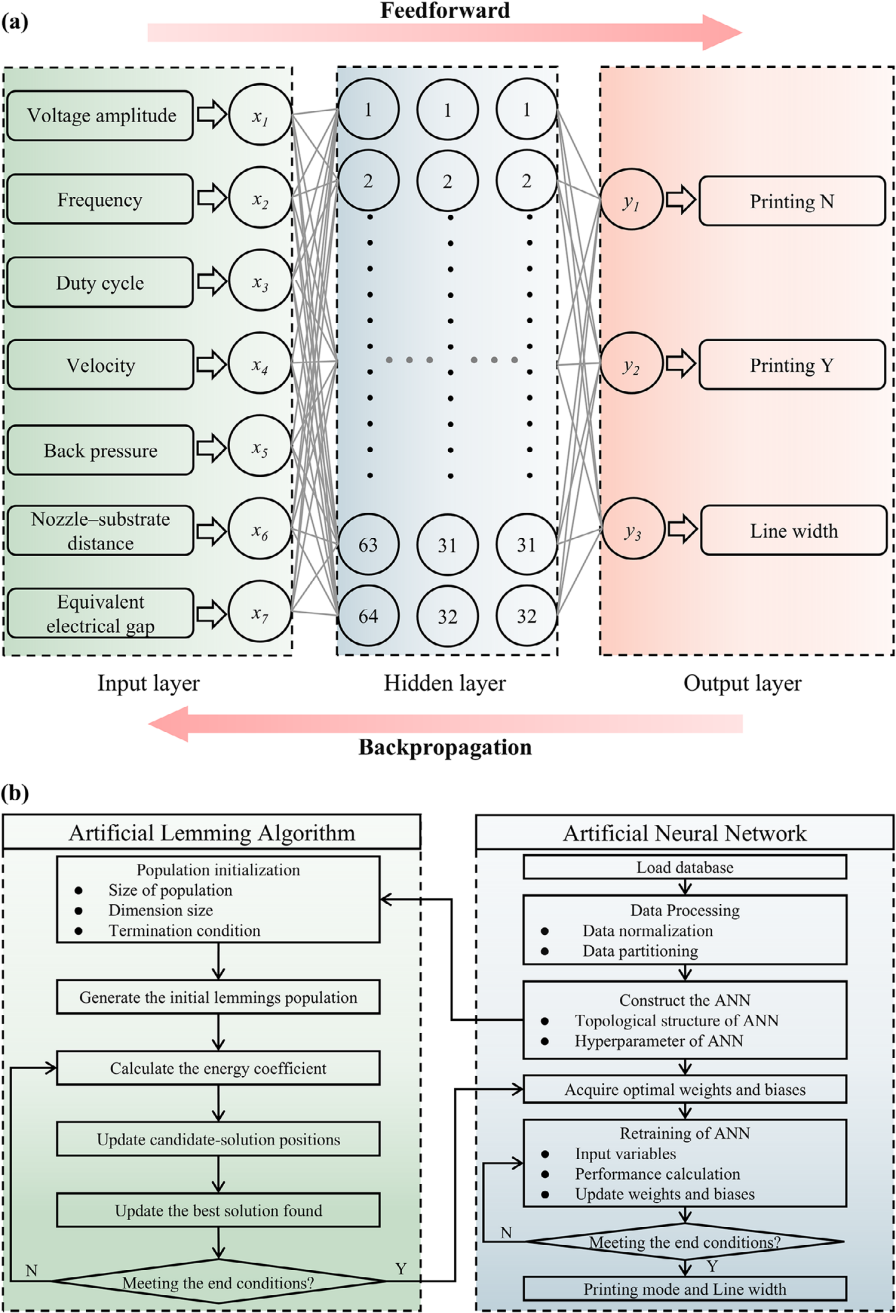
Printing states and line width dimensions prediction model based on ALA‐ANN. (a) network topology, Printing N: a non‐conforming printing state, Printing Y: a favorable printing state; (b) flowchart of ALA‐ANN.

Although ANN can learn non‐linear mapping relationships between input features and outputs by simulating biological neural systems, their performance in practical applications is highly susceptible to the initial settings of weights and biases [52, 53, 54]. This susceptibility may cause the networks to converge at local optima, thus restricting their learning accuracy and generalization capabilities [55, 56]. The ALA effectively identifies optimal solutions to complex problems by mimicking the behavior of lemmings in nature. Therefore, in Stage I of the ML‐AE‐EHD printing approach for curved insulating surfaces, the ALA is introduced to optimize the weights and biases of the ANN. This action enhances the network's learning ability and generalization performance. As a result, an ALA‐ANN model is constructed to predict the printing states and line width dimensions. Figure 2b demonstrates a process for predicting printing states and line widths based on the ALA‐ANN. The diagram portrays the entire workflow, starting from the input of process parameters and equivalent electrical gap, the regulation of network weights and biases using the ALA, followed by network training, and finally leading to the output of prediction results. The detailed parameter settings of the ALA used for ANN optimization are shown in Table S2.

As shown in Figure 3a, after the construction of the ALA‐ANN model for predicting printing states and line width dimensions, the ML‐AE‐EHD printing approach moves on to the phase of initial parameter determination and path parameter regulation. In Stage II, the printing process parameters that meet the target line width requirements at the curved surface printing initiation point are first determined. In Stage III, the equivalent electrical gap at each discrete position along the curved‐surface printing path is calculated. By combining this calculation with the initial process parameters obtained in Stage II, adaptive intelligent regulation of the process parameters along the curved‐surface path is carried out, with the equivalent electrical gap at each position serving as the constraint condition. This helps to alleviate the impact of electric field strength variations on line width consistency. Figure 3b depicts the optimization process for determining the process parameters corresponding to the target line width at the initial position based on the ALA. Figure 3c illustrates the specific procedure for the dynamic and intelligent adjustment of process parameters during printing on the curved surface using the ALA. This is accomplished by integrating the equivalent electrical gap at each position point along the curved surface printing path with the known initial process parameters.

**FIGURE 3:**
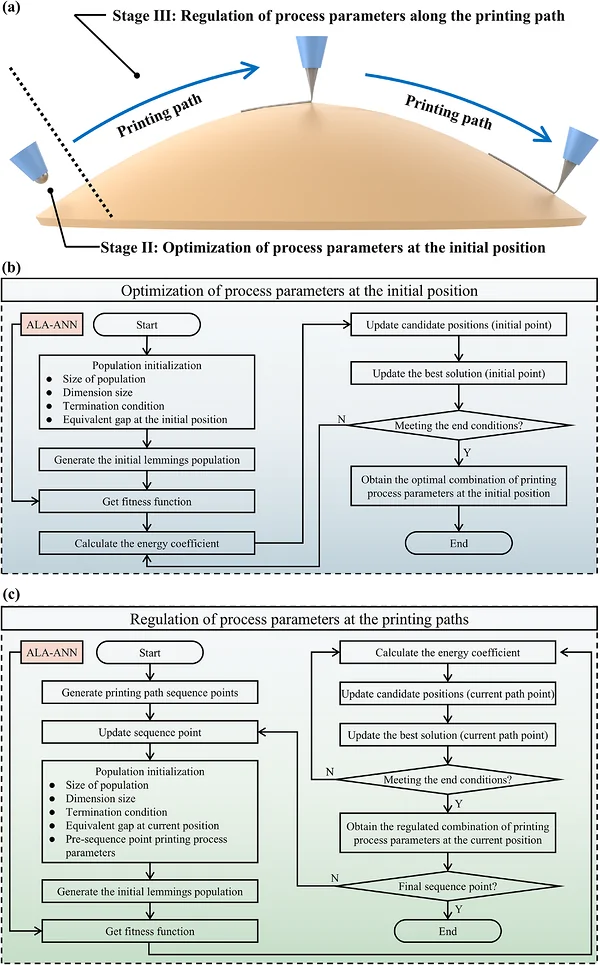
Parameter regulation within the ML‐AE‐EHD printing approach. (a) schematic of multi‐stage parameter regulation for printing on curved substrates; (b) flowchart of optimization of process parameters at the initial position; (c) flowchart of regulation of process parameters at the printing paths.

## 4 Results and Discussion

To more comprehensively assess the ALA‐ANN model's capability to identify printing states and predict line width dimensions during Stage I of the ML‐AE‐EHD printing approach for curved surfaces, this work additionally introduces the traditional ANN, the genetic algorithm‐optimized artificial neural network (GA‐ANN) [57], and the particle swarm optimization‐optimized artificial neural network (PSO‐ANN) [58] as comparison models.

Figure 4 shows the confusion matrices [59] for the different models. As depicted in Figure 4a, the baseline ANN model can already differentiate between the two printing states to a certain degree. However, there are still obvious cases of misclassification. In the all dataset, the ANN model achieves recognition accuracies of 87.57% and 90.19% for favorable and poor printing states, respectively. This indicates that there is still room for improvement in the model's ability to characterize the boundaries of printing states under complex process parameter conditions. After optimization using swarm intelligence algorithms, the model's ability to discriminate between the two types of printing states is enhanced. As shown in Figure 4b, the GA‐ANN model attains recognition accuracies of 95.48% and 93.26% for favorable and poor printing states, respectively, on the all dataset. As shown in Figure 4c, the PSO‐ANN model achieves recognition accuracies of 93.79% and 92.55% in the all dataset, respectively, which also outperform the baseline ANN model. This suggests that optimizing the network's initial weights and biases can boost model performance and improve the ability to distinguish between printing states. In contrast, Figure 4d shows that the ALA‐ANN model exhibits superior classification results across diverse datasets. On the training set, the ALA‐ANN attains recognition accuracies of 98.13% and 95.42% for favorable and poor printing states, respectively. On the test set, the recognition accuracy for these two states is 95.40% and 93.90%, respectively, and the overall recognition accuracy is 94.33%. On the all dataset, they reach 97.46% and 95.04%, respectively. All these accuracies are the highest or near‐highest among the four models.

**FIGURE 4:**
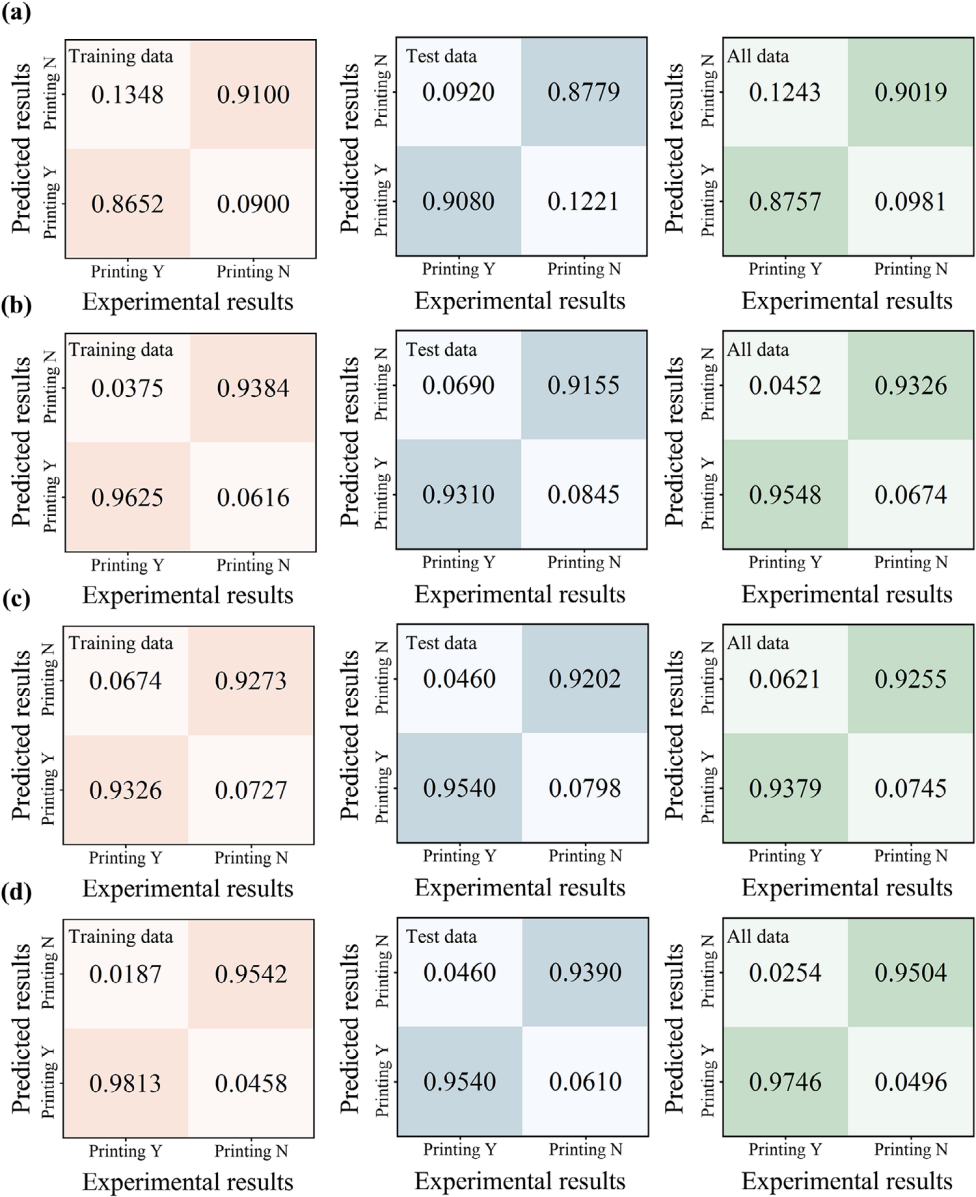
Confusion matrix of prediction models. (a) ANN; (b) GA‐ANN; (c) PSO‐ANN; (d) ALA‐ANN.

Figure 5 presents a comparison of the measured and predicted values for the ANN, GA‐ANN, PSO‐ANN, and ALA‐ANN models in the prediction of line width. All line‐width regression analyses and the corresponding regression metrics reported in this work are based exclusively on favorable printing state samples (Printing Y). As depicted in Figure 5a, the distribution of the predicted points of the ANN model is relatively dispersed. The R^2^ (coefficient of determination, Equation S8) [60] values for the training and testing sets are 0.8358 and 0.8328, respectively, which suggests that the overall prediction accuracy is limited. As shown in Figure 5b, after optimization using swarm intelligence algorithms, the R^2^ values of the GA‐ANN model increased by 0.0710 and 0.0437 on the training and test datasets, respectively. Meanwhile, as shown in Figure 5c, the R^2^ values of the PSO‐ANN model increased by 0.0643 and 0.0478, respectively. Moreover, the predicted points are more concentrated around the ideal fit line. In contrast, as shown in Figure 5d, the ALA‐ANN model exhibits optimal fitting and generalization performance, attaining R^2^ values of 0.9723 and 0.9452 on the training and test datasets, respectively. Moreover, most of the predicted points are within a ±20% error margin, which suggests that the ALA‐ANN model can precisely map the intricate nonlinear relationship between printing process parameters and line width.

**FIGURE 5:**
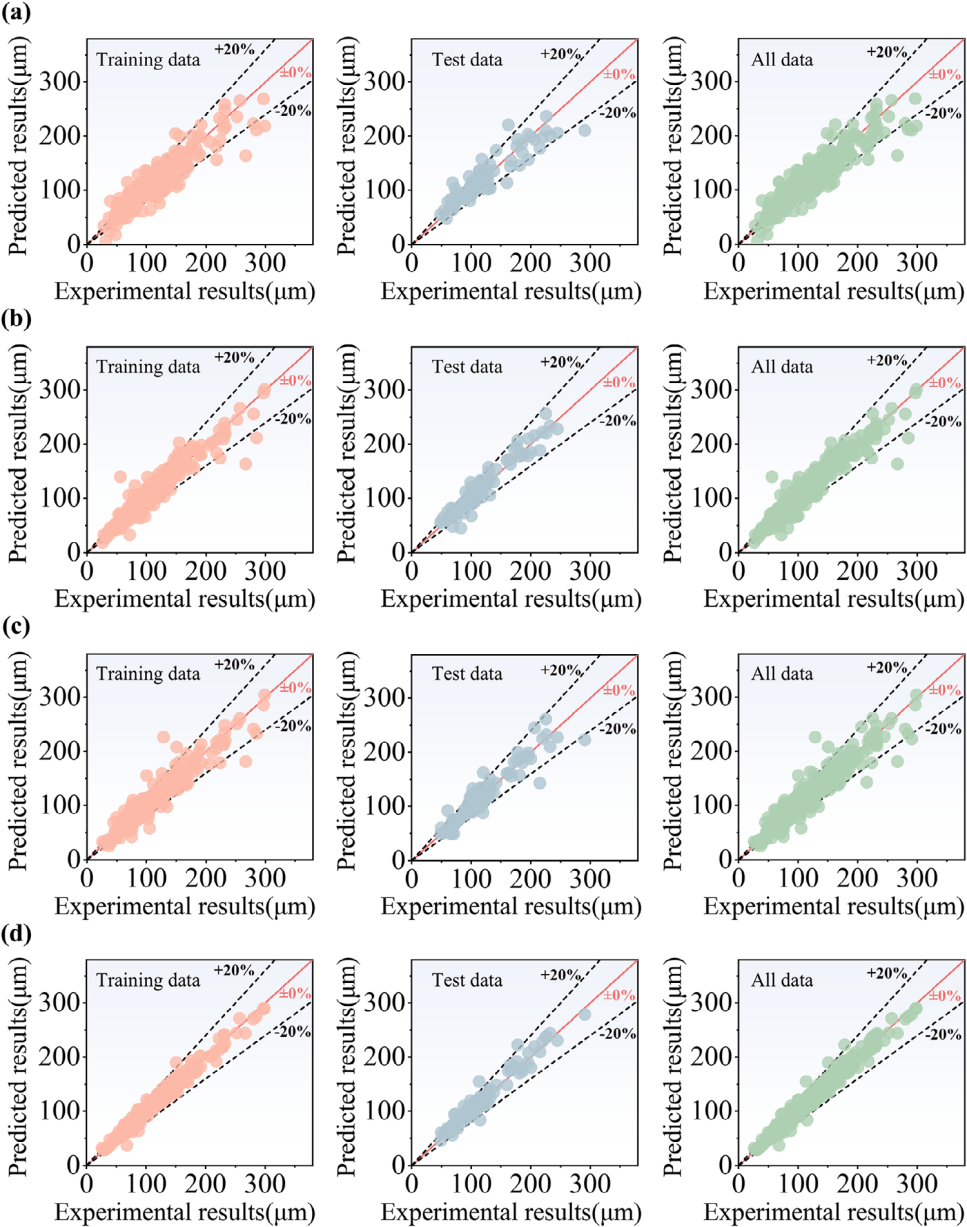
The predictive performance of the models for line width. (a) ANN; (b) GA‐ANN; (c) PSO‐ANN; (d) ALA‐ANN.

Figure 6 depicts the distribution of the absolute relative error (ARE, Equation S9) [61] for the ANN, GA‐ANN, PSO‐ANN, and ALA‐ANN models in line width prediction. The scatter plot, violin plot, and box plot respectively, illustrate the dispersion, probability density, and quantile intervals of the error. As shown in Figure 6a, the mean absolute relative error (MARE, Equation S10) [47] of the ANN, GA‐ANN, PSO‐ANN, and ALA‐ANN models on the training set are 14.41%, 9.81%, 10.37%, and 6.77%, respectively. Figure 6b presents the corresponding error distributions on the test set, with MARE values of 11.97%, 9.74%, 9.90%, and 8.41%, respectively. For the all dataset shown in Figure 6c, the corresponding MARE values are 13.79%, 9.79%, 10.26%, and 7.16%, respectively. Among the four models, the ALA‐ANN model exhibits the lowest MARE and a more concentrated error distribution across all three datasets. Moreover, the errors are mainly concentrated in the lower range, with a significantly reduced distribution width and volatility. Based on the above results, the ALA‐ANN model is capable of accurately predicting the printing states and line width dimensions on curved insulating surfaces, thus fulfilling the requirements for subsequent intelligent parameter optimization.

**FIGURE 6:**
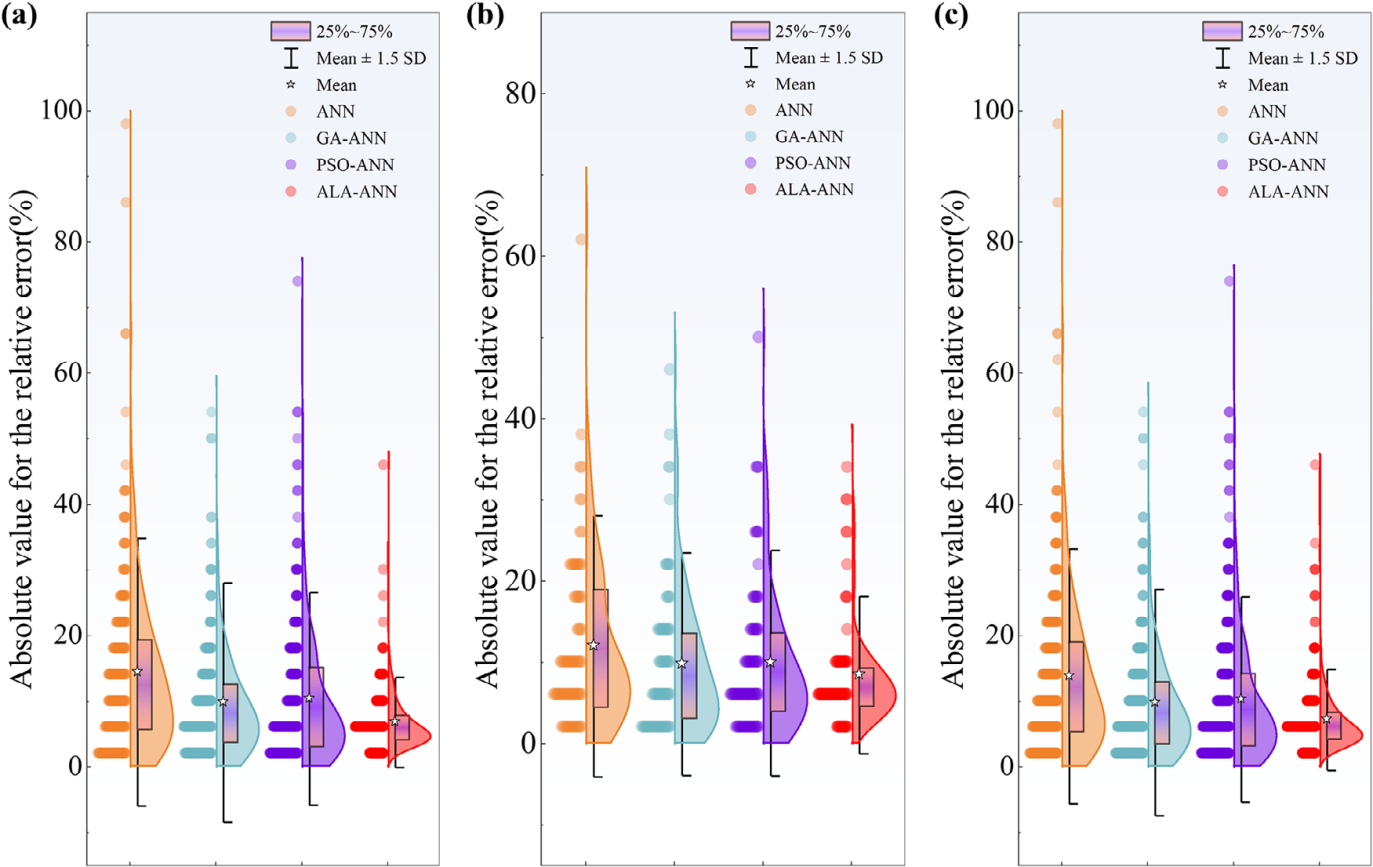
Analysis of line width prediction errors. (a) training data; (b) test data; (c) all data.

Figure 7a–e illustrates the process of determining process parameters in Stage II of the ML‐AE‐EHD printing approach for curved surfaces. At this stage, an iterative optimization search is carried out using the ALA to determine the process parameters at the initial position that satisfy the target line width requirements, gradually converging toward the optimal solution. Figure 7f–h shows the experimental results of printed structures with different target line widths at the initial position using the optimized printing process parameters. Figure 7i conducts a further statistical analysis of the relative error between the actual printed line width and the target dimension at the initial position. When the target line widths are 30, 60, and 90 µm, respectively, the corresponding average relative errors are 10.24%, 6.77%, and 5.21%. The results presented above demonstrate that at the initial position, ALA can precisely optimize the printing process parameters corresponding to the target line width under the constraints of a stable printing state. This offers a reliable and feasible basis for intelligent parameter control along curved surfaces printing paths in the subsequent Stage III.

**FIGURE 7:**
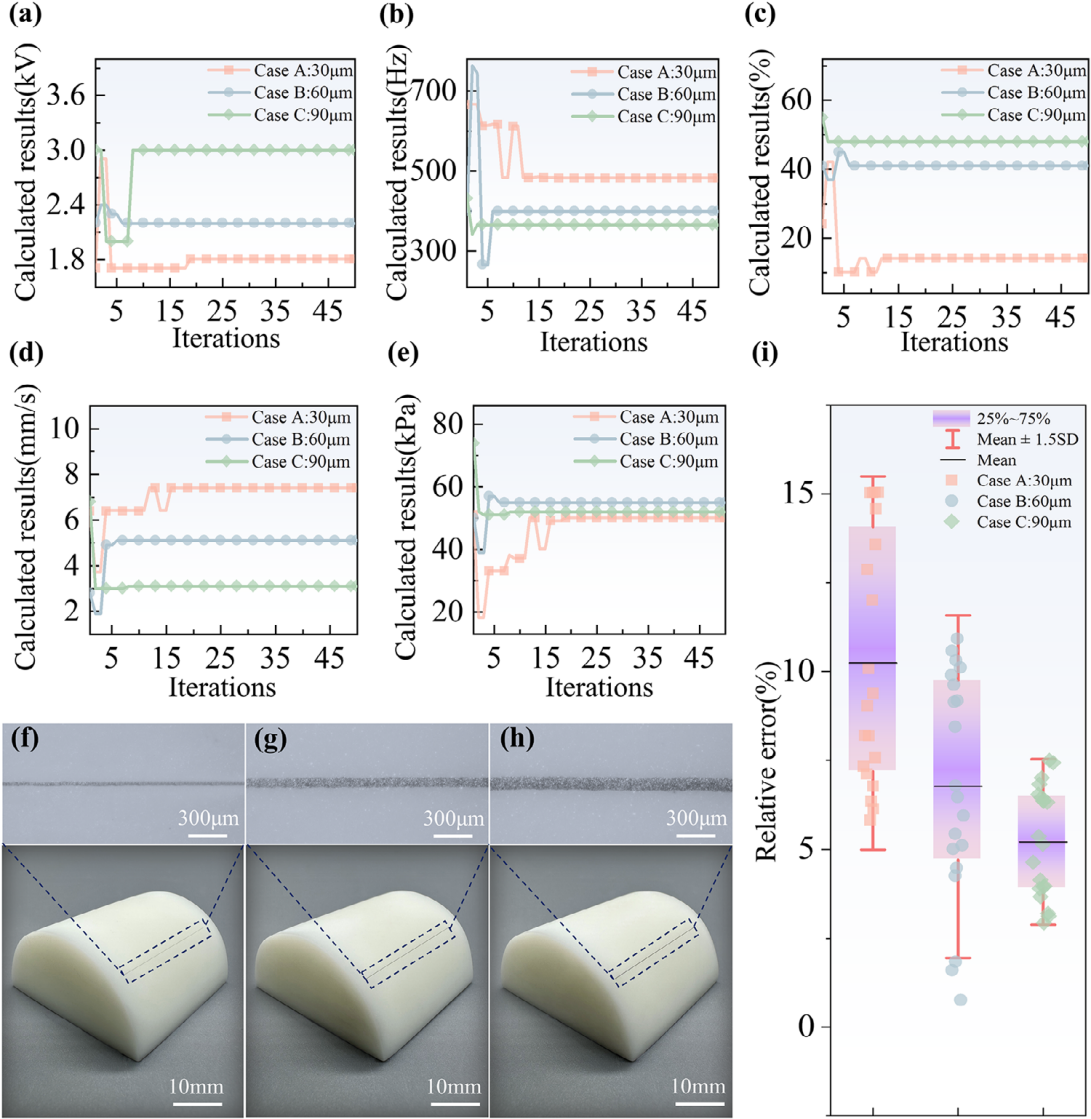
Optimization process parameters for initial positioning. (a) convergence curve of voltage amplitude; (b) convergence curve of frequency; (c) convergence curve of duty cycle; (d) convergence curve of velocity; (e) convergence curve of back pressure; (f) 30µm line width; (g) 60µm line width; (h) 90µm line width; (i) relative errors in three cases.

After acquiring the printing process parameters that satisfy the target line width requirements at the initial position, the equivalent electrical gap at each discrete position point along the printing path on the curved substrate is further computed. At each position point, the corresponding process parameters for the target line width are ascertained based on the ALA. Based on the solution results from each position point, the process parameters are dynamically regulated along the path during the printing process to compensate for the electric field variations caused by changes in the equivalent electrical gap. This mitigates the negative impacts on the consistency of the line width.

Figure 8a–e shows the process parameter regulation curves on the curved surfaces when the nozzle position changes in Stage III of the ML‐AE‐EHD approach. Across the three target line‐width cases, the voltage amplitude and printing velocity remain relatively stable along the printing path, whereas the frequency, duty cycle, and back pressure generally exhibit more pronounced position‐dependent adjustments. For the target line widths of 30 and 90 µm, the frequency generally decreases toward the middle portion of the path and subsequently increases, whereas it generally decreases with a slight recovery near the end of the path for 60 µm. The duty cycle generally decreases for the target line widths of 30 and 60 µm but remains nearly constant for 90 µm. The back pressure fluctuates slightly for 30 µm, generally decreases for 60 µm, and remains at a slightly lower level over the middle portion of the path before returning to its initial level near the end for 90 µm. These parameter adjustments indicate that the model coordinates the process parameters according to the printing position and target line width to compensate for the influence of position‐dependent electric‐field variations on the printing results.

**FIGURE 8:**
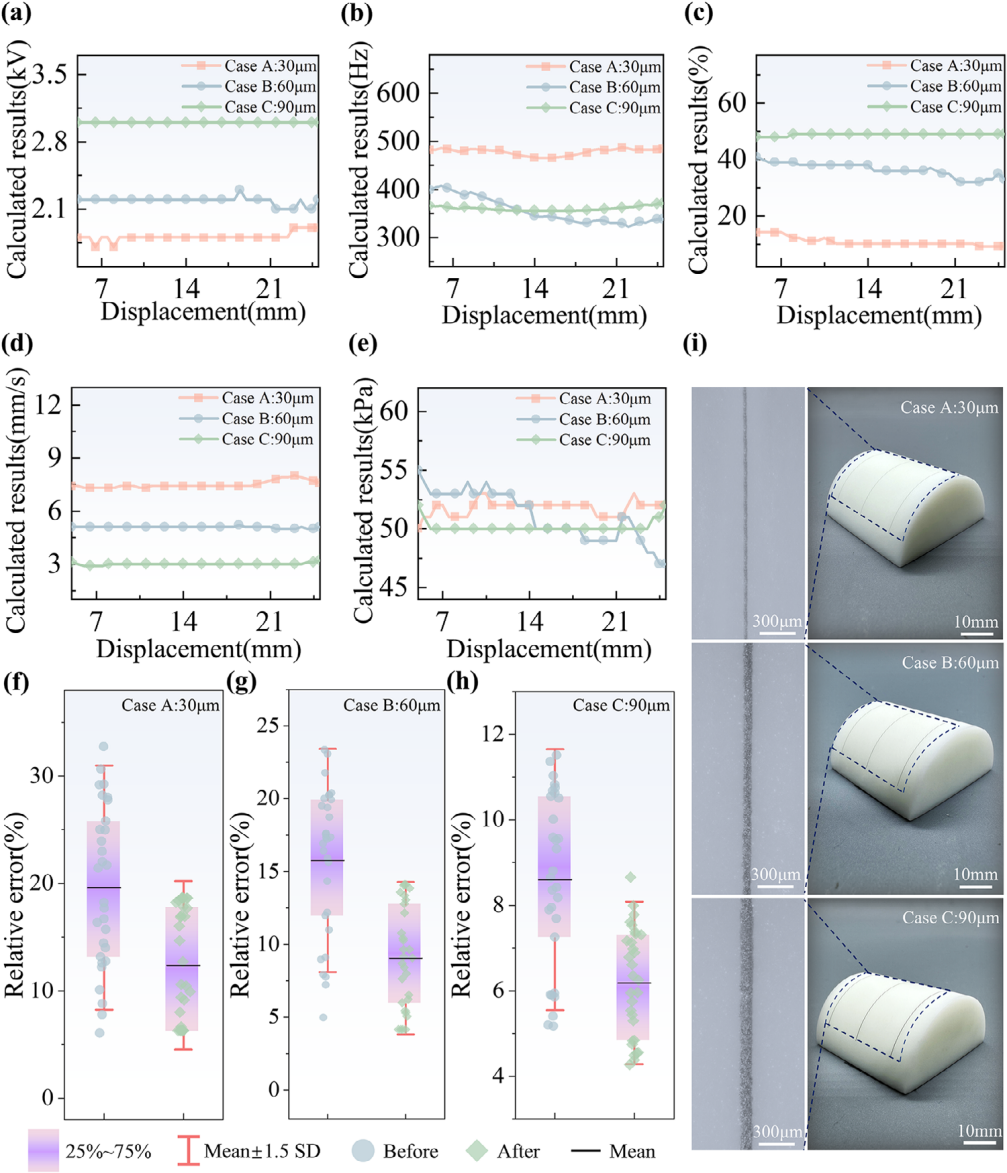
Process parameter regulation on curved surfaces. (a) regulation curve of voltage amplitude; (b) regulation curve of frequency; (c) regulation curve of duty cycle; (d) regulation curve of velocity; (e) regulation curve of back pressure; (f) relative error for 30 µm line width; (g) relative error for 60 µm line width; (h) relative error for 90 µm line width; (i) curved‐surface conformal lines with varying widths.

Along the semicylindrical printing path, the positions corresponding to path displacements of 5, 15, and 25 mm are located in a lower region on one side, at the apex, and in a lower region on the opposite side, respectively. Although the nozzle‐substrate distance is maintained constant, the local substrate height and equivalent electrical gap increase from the lower region to the apex and subsequently decrease toward the opposite side. The larger equivalent electrical gap near the apex corresponds to a weaker local driving electric field under otherwise identical electrical conditions, thereby requiring the process parameters to be adjusted accordingly. Taking the target line width of 90 µm as an example, the parameter combinations of voltage amplitude, frequency, duty cycle, printing velocity, and back pressure at these three representative positions are 3.0 kV, 366 Hz, 48%, 3.1 mm/s, and 52 kPa; 3.0 kV, 356 Hz, 49%, 3.0 mm/s, and 50 kPa; and 3.0 kV, 371 Hz, 49%, 3.2 mm/s, and 52 kPa, respectively. The generally consistent parameter combinations at the two lower positions, combined with the corresponding adjustments at the apex, demonstrate that the model coordinates multiple process parameters based on local geometric conditions and equivalent electrical gaps.

Figure 8f–h presents the comparison of the printed line width results before and after parameter regulation. The experimental results demonstrate that, in comparison with the unregulated conditions, the average relative deviation decreased by 7.24 percentage points, 6.70 percentage points, and 2.41 percentage points when the target line widths are 30, 60, and 90 µm, respectively. The line‐width consistency and repeatability across the curved surface are further evaluated in Figure S2 and Table S3. The curved‐surface printing process and deposited‐line morphology are presented in Figure S3. Figure 8i illustrates a diverse range of target line width structures achieved through the intelligent regulation on the curved insulating surface. In summary, the intelligent regulation of the ML‐AE‐EHD printing approach for curved surfaces effectively suppresses the line width fluctuations caused by the variations in electric field conditions along curved printing paths, thereby enabling high‐precision and stable printing of different target line widths on curved insulating substrates.

Figure 9a–e shows the precise patterning results obtained with different line width dimensions on various types of curved substrates by using the ML‐AE‐EHD printing approach. During micro pattern printing, the equivalent electrical gap at each discrete position along the path is initially calculated. Subsequently, based on the ALA, the process parameters are dynamically adjusted along the path to offset the influence of variations in the equivalent electrical gap on the printing states and line‐width dimensions. Figure 9a,c–e represent solid curved‐surface substrates, whereas Figure 9b illustrates thin‐walled hollow curved‐surface structures. Despite the variations in structural type and curvature distribution among different curved surface substrates, the printed lines maintain favorable continuity and dimensional stability on all substrates, and no discernible printing state instability or line width drift is observed. The results clearly demonstrate that the proposed ML‐AE‐EHD printing approach allows for adaptive control of printing parameters on various complex curved substrates, and it can consistently achieve the target line width. This offers reliable process support for the precision manufacturing of conformal micro‐patterns on curved substrates.

**FIGURE 9:**
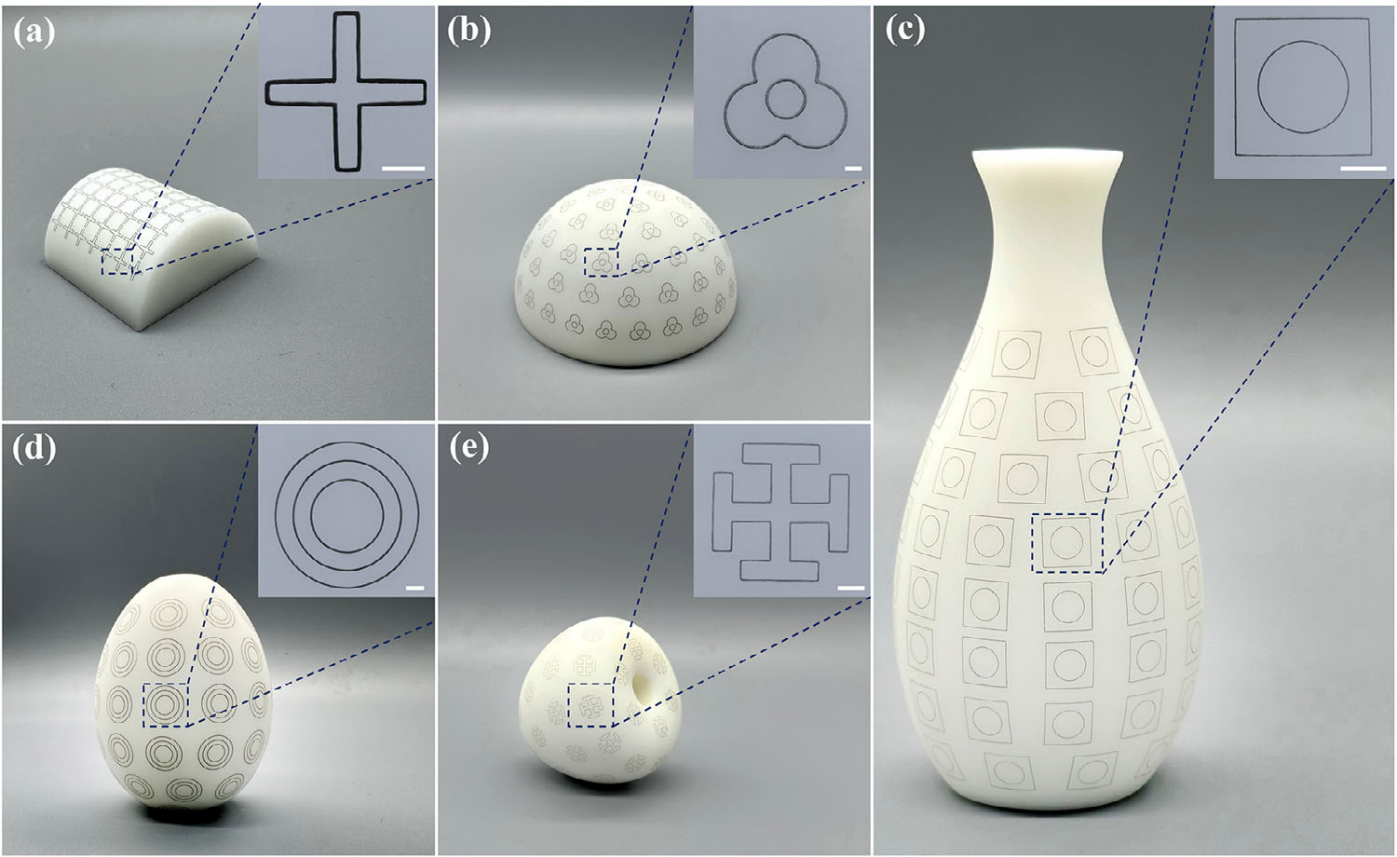
Conformal microstructures with varying line widths printed on different surfaces. (a) semicircular surface; (b) hemispherical surface; (c) vase‐shaped surface; (d) ovoid surface; (e) apple‐shaped surface. Scale bars, 1 mm.

To further validate the capability of the ML‐AE‐EHD printing approach in ensuring dimensional accuracy and performance of printed microstructures, Figure 10 presents the printed results for a curved frequency‐selective surface structure alongside its electromagnetic performance test outcomes. Figure 10a showcases the design dimensions of the curved‐surface FSS (detailed dimensions are presented in Table S4), whereas Figure 10b portrays the structure fabricated through the ML‐AE‐EHD printing approach on the curved substrate. To evaluate the dimensional accuracy of the printed curved‐surface FSS, line‐width measurements are conducted at 100 locations randomly selected throughout the printed resonant microstructure array. The measured line widths have a mean value of 150.09 µm and a standard deviation of 3.47 µm. The mean absolute error relative to the designed line width is 2.65 µm, and 96% of the measurements fall within the range of 145–155 µm. These results demonstrate good line‐width accuracy and dimensional consistency of the printed curved‐surface FSS. A detailed statistical analysis of the line‐width measurements is provided in Figure S4. Figure 10c compares the simulated and measured S21 (transmission coefficient) [28] curves. The simulated and measured resonant frequencies are 6.864 GHz and 7.114 GHz, respectively, corresponding to a relative deviation of 3.64%. The corresponding minimum S21 values are −43.55 and −35.35 dB, respectively, with a relative deviation of 18.83%. Overall, the simulated and measured electromagnetic responses exhibit a high degree of consistency. The dimensional accuracy of the printed structure and the agreement between the simulated and measured electromagnetic responses jointly demonstrate the capability of the ML‐AE‐EHD printing approach for fabricating curved conformal functional structures.

**FIGURE 10:**
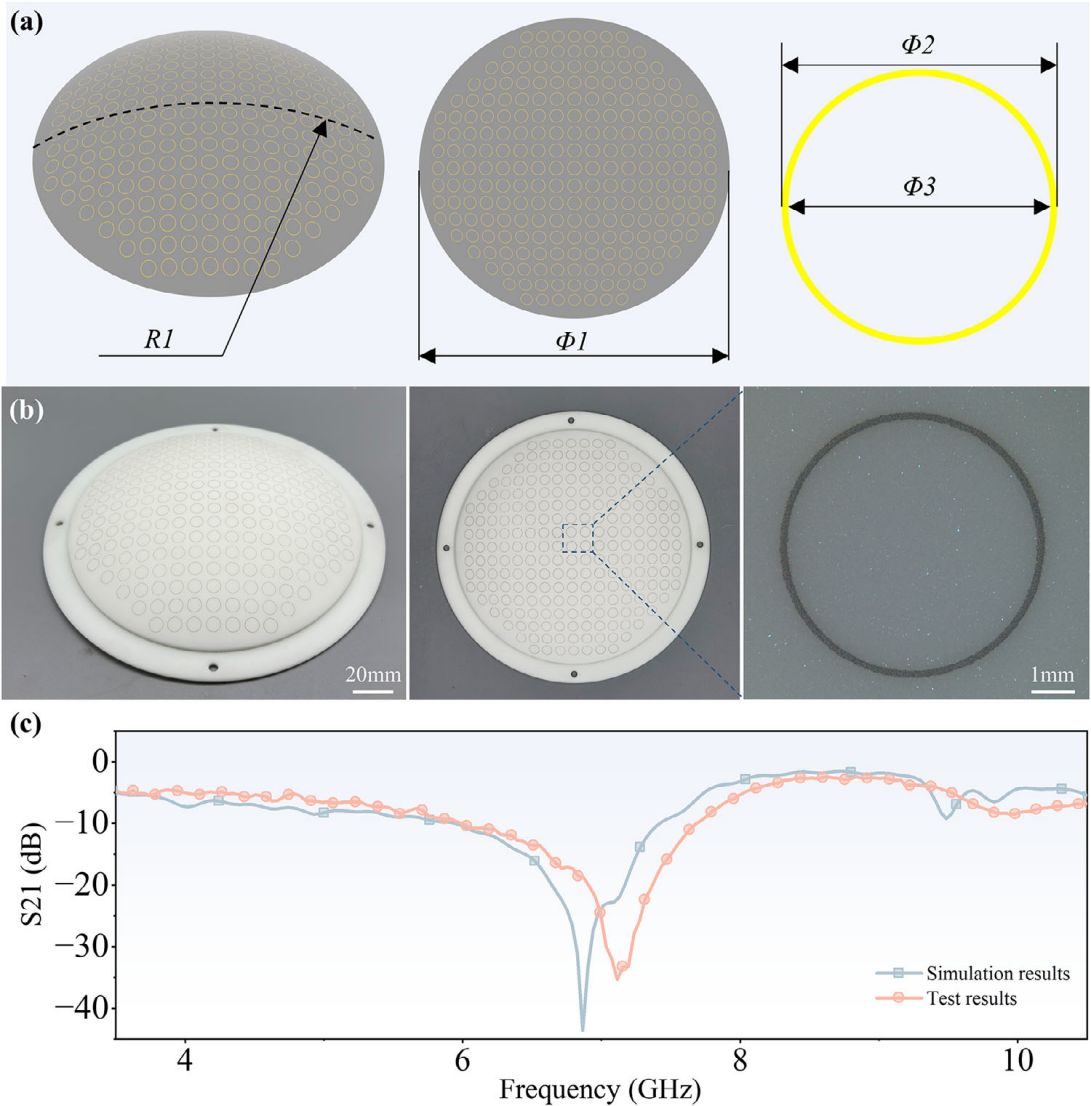
Printed surface‐conformal FSS. (a) design of the curved‐surface FSS; (b) printed FSS sample; (c) comparison of S21 simulation and test result.

Further evidence for the broad applicability of the ML‐AE‐EHD printing approach in surface‐conformal functional structures is provided by the fabrication of a surface‐conformal coplanar waveguide (CPW) broadband antenna [62]. Figure 11 presents the fabrication and testing results of the antenna. Figure 11a shows the schematic design of the antenna structure, with the detailed dimensions provided in Table S5, and Figure 11b presents the antenna fabricated using the ML‐AE‐EHD printing approach. Figure 11c compares the simulated and measured S11 (reflection coefficient) curves [63]. The simulated and measured main resonant frequencies are 5.800 GHz and 5.960 GHz, respectively, corresponding to a relative deviation of 2.76%. The corresponding minimum S11 values are −40.15 and −38.04 dB, respectively, corresponding to a relative deviation of 5.26%. Within the frequency range of 3–8 GHz, the measured S11 remains below or close to −10 dB. Figure 11d presents the corresponding voltage standing wave ratio (VSWR) curves [64], with the measured VSWR remaining below 2 throughout this frequency range. Overall, the simulated and measured electromagnetic responses exhibit a high degree of consistency, confirming that the printed antenna achieves the designed electromagnetic performance. These results further demonstrate the applicability of the ML‐AE‐EHD printing approach to the high‐precision fabrication of functional structures on curved insulating substrates.

**FIGURE 11:**
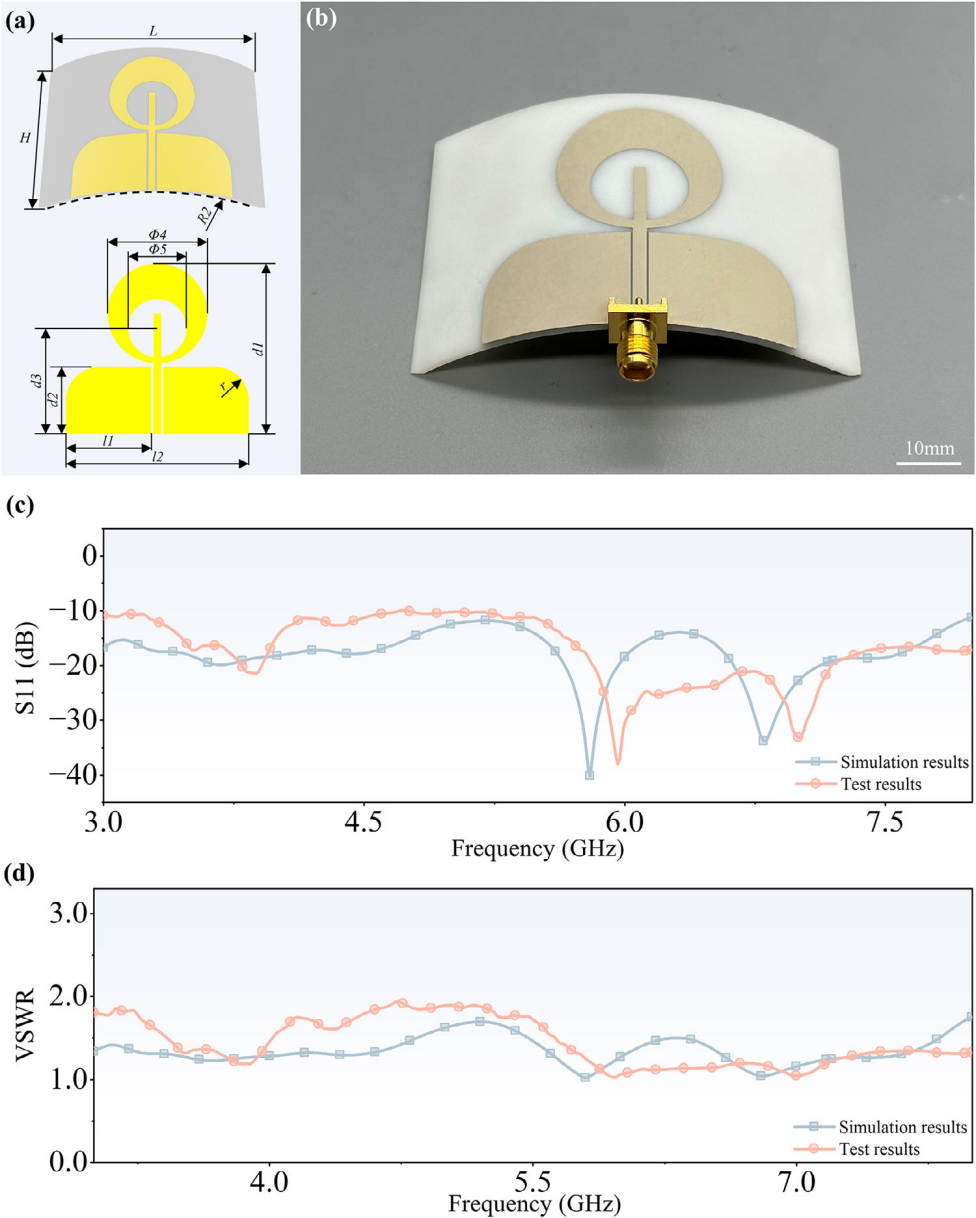
Printed surface‐conformal antenna. (a) design of the antenna; (b) printed antenna sample; (c) comparison of S11 simulation and test result; (d) comparison of VSWR simulation and test result.

## 5 Conclusions

To achieve high‐precision fabrication of conformal functional structures on curved insulating substrates for curved conformal electronics, this work presents an intelligent field‐adaptive EHD printing theoretical and technical framework to address local electric‐field fluctuations during curved‐surface printing. By integrating alternating‐electric‐field modulation for reducing residual‐charge effects, an equivalent electrical gap for characterizing path‐dependent electric‐field conditions, and machine learning based parameter regulation for compensating position‐dependent field variations, this framework gives rise to the ML‐AE‐EHD printing approach, thereby enabling stable and controllable deposition and the high‐precision fabrication of conformal functional structures on curved insulating substrates.

In Stage I of the ML‐AE‐EHD printing approach, the alternating electric field enables periodic charge neutralization, thereby alleviating the influence of residual charges on the driving electric field, suppressing the whipping behavior of the liquid bridge, and improving the printing stability. On this basis, a high‐precision prediction model for the printing state and line width dimensions is established. The results indicate that the model shows outstanding performance across diverse datasets. On the test set, the recognition accuracy for the printing state attains 94.33%, and the R^2^ between the predicted line width and the actual results reaches 0.9452, with a mean absolute relative error of 8.41%. These results verify the predictive accuracy and robustness of the model for both the printing state and line width dimensions.

In Stage II of the ML‐AE‐EHD printing approach, the printing process parameters corresponding to the target line width at the initial position on the curved surface are optimized through ALA‐based inverse search. The experimental results show that the average relative errors for target line widths of 30, 60, and 90 µm are 10.24%, 6.77%, and 5.21%, respectively. This confirms the effectiveness of the proposed approach in inversely optimizing process parameters for target‐width fabrication at the initial position on curved substrates.

In Stage III of the ML‐AE‐EHD printing approach, the process parameters are intelligently regulated along the curved‐surface printing path according to variations in the equivalent electrical gap. By comparing the actual line widths on curved substrates before and after regulation, the average relative errors for target line widths of 30, 60, and 90 µm are found to decrease by 7.24 percentage points, 6.70 percentage points, and 2.41 percentage points, respectively. These results confirm the effectiveness of the proposed approach in achieving line width control during printing on curved insulating substrates. Based on this approach, delicate patterns with varying line widths are successfully printed on diverse curved insulating substrates, and the curved conformal FSS and CPW antenna with excellent electromagnetic performance are successfully fabricated. These results demonstrate that the ML‐AE‐EHD printing approach enables stable and precise fabrication of conformal structures on curved insulating substrates, while further validating the effectiveness of the proposed intelligent field‐adaptive EHD printing framework. This work provides a systematic theoretical and technical foundation for improving the manufacturing capability of conformal electronics and promoting their further development.

## Author Contributions

**Chang Liu**: conceptualization, investigation, writing – original draft, methodology, visualization, writing – review and editing. **Yiwen Feng**: conceptualization, investigation, writing – original draft, methodology, visualization, writing – review and editing. **Xu Chen**: data curation. **Kaiwen Chen**: data curation. **Ran Zhang**: data curation. **Xiaopeng Zhang**: data curation. **Liujia Suo**: data curation. **Xiangji Chen**: data curation. **Zefei Li**: data curation. **Fengshu Wang**: data curation. **Ruixuan Xu**: software. **Shuaiwu Liu**: software. **Shiwen Liang**: data curation. **Lingjie Kong**: software. **Dazhi Wang**: conceptualization, investigation, validation.

## Conflicts of Interest

The authors declare no conflicts of interest.

## Supporting information




**Supporting File**: advs78343‐sup‐0001‐SuppMat.pdf.

## Data Availability

The data that support the findings of this study are available from the corresponding author upon reasonable request.
