# Supplementary material for: Intelligent Field‐Adaptive Electrohydrodynamic Printing for Curved Conformal Electronics
Source: Adv Sci (Weinh). 2026 Oct 18:e78343. Online ahead of print. doi: 10.1002/advs.78343 (PMC13677044; doi:10.1002/advs.78343)
Supplement: Supplementary file 1 [file ADVS-9999-e78343-s001.pdf]

# Supporting Information

## Intelligent Field-Adaptive Electrohydrodynamic Printing for Curved Conformal Electronics

*Chang Liu[1#], Yiwen Feng[1,2#*], Xu Chen[1], Kaiwen Chen[1], Ran Zhang[1,2,3], Xiaopeng Zhang[4], Liujia Suo[1], Xiangji Chen[1], Zefei Li[1], Fengshu Wang[1], Ruixuan Xu[1], Shuaiwu Liu[2], Shiwen Liang[3], Lingjie Kong[3] and Dazhi Wang[1,2,3*]*

# 1. Fitness functions for process-parameter inversion and regulation

For each candidate process-parameter vector $\boldsymbol{x}$, the ANN outputs the probabilities $p_{\mathrm{Y}}(\boldsymbol{x})$ and $p_{\mathrm{N}}(\boldsymbol{x})$ for Printing Y and Printing N, respectively, together with the predicted line width $\hat{y}(\boldsymbol{x})$.

The normalized line-width deviation is defined as:

$$e_y(\boldsymbol{x}) = \frac{|\hat{y}(\boldsymbol{x}) - y_t|}{|\hat{y}(\boldsymbol{x}) - y_t| + y_t} \tag{S1}$$

where $y_t$ is the target line width.

The fitness function used in **Stage II** is:

$$F_{\mathrm{II}}(\boldsymbol{x}) = \begin{cases} 0.2[1 - p_{\mathrm{Y}}(\boldsymbol{x})] + 0.8e_y(\boldsymbol{x}), & p_{\mathrm{Y}}(\boldsymbol{x}) > p_{\mathrm{N}}(\boldsymbol{x}) \\ 1 + p_{\mathrm{N}}(\boldsymbol{x}) - p_{\mathrm{Y}}(\boldsymbol{x}), & p_{\mathrm{Y}}(\boldsymbol{x}) \le p_{\mathrm{N}}(\boldsymbol{x}) \end{cases} \tag{S2}$$

In **Stage III**, the normalized variation in the process parameters between two adjacent printing-path points is defined as:

$$e_p\left(\boldsymbol{x}_i, \boldsymbol{x}_{i-1}\right) = \sqrt{\frac{1}{m}\sum_{j=1}^{m}\left(\frac{x_{i,j} - x_{i-1,j}}{x_{j,\max} - x_{j,\min}}\right)^2} \tag{S3}$$

Here, $\boldsymbol{x}_i$ and $\boldsymbol{x}_{i\text{-}1}$ denote the process-parameter vectors at the current and preceding printing-path points, respectively; $x_{i,j}$ is the $j$-th process parameter at the current point; $\mathrm{x}_{j,\max}$ and $\mathrm{x}_{j,\min}$ are its upper and lower bounds; and $m$ = 5 is the number of optimized process parameters.

The fitness function used in **Stage III** is

$$F_{\mathrm{III}}\left(\boldsymbol{x}_i\right) = \begin{cases} 0.2\left[1 - p_{\mathrm{Y}}\left(\boldsymbol{x}_i\right)\right] + 0.6e_y\left(\boldsymbol{x}_i\right) + 0.2e_p\left(\boldsymbol{x}_i, \boldsymbol{x}_{i-1}\right), & p_{\mathrm{Y}}\left(\boldsymbol{x}_i\right) > p_{\mathrm{N}}\left(\boldsymbol{x}_i\right) \\ 1 + p_{\mathrm{N}}\left(\boldsymbol{x}_i\right) - p_{\mathrm{Y}}\left(\boldsymbol{x}_i\right), & p_{\mathrm{Y}}\left(\boldsymbol{x}_i\right) \le p_{\mathrm{N}}\left(\boldsymbol{x}_i\right) \end{cases} \tag{S4}$$

Here, 1-$p_{\mathrm{Y}}$ represents the classification-confidence term, $e_y$ represents the normalized line-width deviation, and $e_p$ represents the normalized process-parameter variation. When $p_{\mathrm{Y}} > p_{\mathrm{N}}$, the candidate is classified as Printing Y and its line-width deviation is evaluated. Otherwise, the candidate is classified as Printing N and only the classification penalty is calculated. Both fitness functions are minimized during optimization.

## 2. Evaluation of process-parameter sampling adequacy and space-filling characteristics

To quantitatively evaluate whether the 1200 sampled process-parameter combinations provide adequate and uniform coverage of the seven-dimensional process-parameter space, the sample distribution is analyzed using the centered $L_2$-discrepancy and nearest-neighbor distance. The original process-parameter vector of the $i$th sample point is expressed as $\boldsymbol{x}_i$. To eliminate dimensional differences among the process parameters, each component is normalized to the interval [0,1]:

$$u_{i,j} = \frac{x_{i,j} - x_{j,\min}}{x_{j,\max} - x_{j,\min}} \tag{S5}$$

where $x_{i,j}$ and $u_{i,j}$ denote the original and normalized values of the $j$th process parameter for the $i$th sample point, respectively, and $x_{j,\max}$ and $x_{j,\min}$ are the corresponding lower and upper bounds.

The centered $L_2$-discrepancy is used to quantify the overall deviation of the sample distribution from an ideal uniform distribution:

$$\begin{cases} A_i = \prod_{j=1}^{n} \left[ 1 + \frac{1}{2}\left| u_{i,j} - \frac{1}{2} \right| - \frac{1}{2}\left| u_{i,j} - \frac{1}{2} \right|^2 \right] \\ B_{ik} = \prod_{j=1}^{n} \left[ 1 + \frac{1}{2}\left| u_{i,j} - \frac{1}{2} \right| + \frac{1}{2}\left| u_{k,j} - \frac{1}{2} \right| - \frac{1}{2}\left| u_{i,j} - u_{k,j} \right| \right] \\ D_{\mathrm{CL2}} = \left( \frac{13}{12} \right)^n - \frac{2}{N}\sum_{i=1}^{N} A_i + \frac{1}{N^2}\sum_{i=1}^{N}\sum_{k=1}^{N} B_{ik} \end{cases} \tag{S6}$$

where $N$ is the total number of sample points and $n$ is the number of process parameters used as inputs to the ANN model. A smaller value of $D_{\mathrm{CL2}}$ indicates a smaller overall deviation from a uniform distribution and better space-filling characteristics. The calculated centered $L_2$-discrepancy of the 1200 normalized sample points is 0.0081, indicating a small overall deviation from a uniform distribution and good space-filling characteristics within the specified seven-dimensional process-parameter space.

The nearest-neighbor distance is further used to evaluate the local spacing among the sample points. For the $i$th sample point, its nearest-neighbor distance is defined as the minimum normalized Euclidean distance to any other sample point:

$$d_i = \min_{\substack{k=1,\ldots,N \\ k\neq i}} \sqrt{\sum_{j=1}^{n}\left(u_{i,j} - u_{k,j}\right)^2} \tag{S7}$$

where $d_i$ denotes the nearest-neighbor distance of the $i$th sample point, and $u_{j,k}$ denotes the normalized value of the $j$th process parameter for the $k$th sample point.

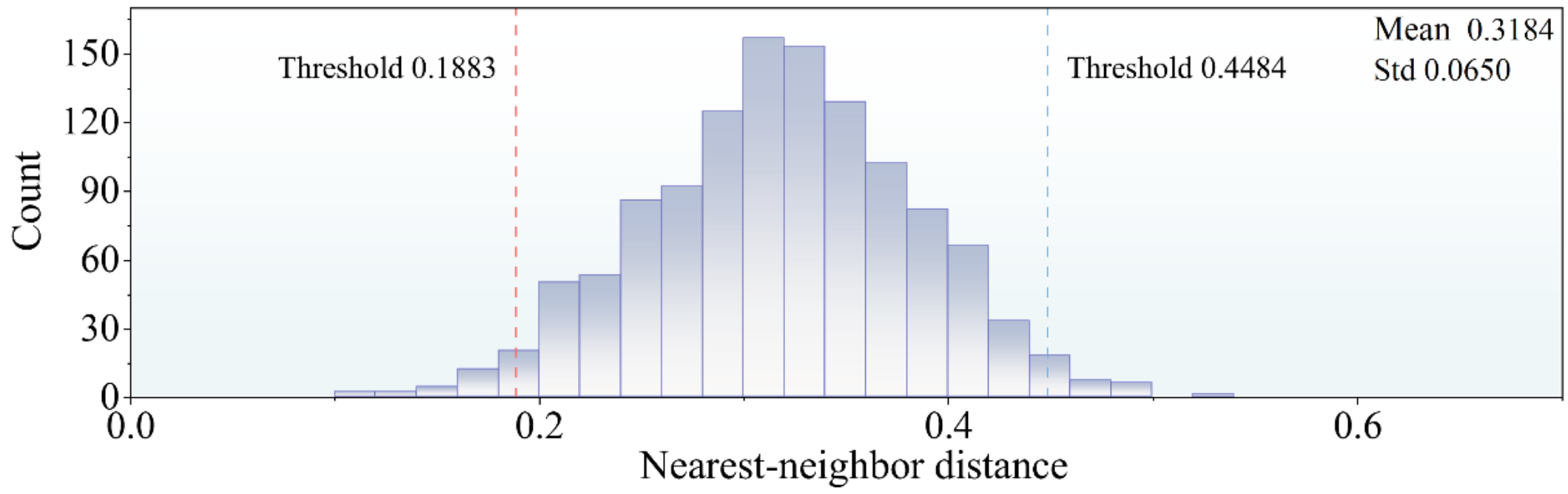


Figure S1 Distribution of the nearest-neighbor distances.

As shown in **Figure S1**, the nearest-neighbor distances exhibit a unimodal distribution concentrated around the mean value. The mean and standard deviation of the nearest-neighbor distances are 0.3184 and 0.0650, respectively. Using the mean plus or minus two standard deviations as reference boundaries, the lower and upper thresholds are determined to be 0.1883 and 0.4484, respectively. Among the 1200 sample points, 1145 points, accounting for 95.42% of the total, have nearest-neighbor distances within this interval. These quantitative results demonstrate that the sample points exhibit good overall space-filling characteristics and relatively balanced local spacing, without obvious clustering or spatial isolation. Therefore, the 1200 sampled process-parameter combinations provide uniform and adequate coverage of the specified seven-dimensional process-parameter space, thereby providing a well-distributed and representative dataset for subsequent model development.

## 3. ANN architecture, training, and optimization settings

To facilitate the reproducibility of the model development, the ANN architecture and related optimization settings are provided in **Tables S1** and **S2**. The ANN consists of seven input neurons, three hidden layers, and three output neurons, with 64, 32, and 32 neurons in the respective hidden layers. The ANN is trained using the Adam optimizer, whereas the ALA is employed to optimize the initial weights and biases of the ANN. The detailed ANN architecture and training settings are summarized in **Table S1**, and the corresponding ALA settings are listed in **Table S2**.

Table S1 Hyperparameters of the ANN model.

| Parameters | Values |
| --- | --- |
| Maximum number of epochs to train | 150 |
| Learning rate | 0.01 |
| Number of input neurons | 7 |
| Number of output neurons | 3 |
| Number of hidden layers | 3 |
| Number of neurons in hidden layers | 64-32-32 |
| Optimizer | Adam |
| Decay rate | 0.85 |

Table S2 The parameters of the ALA utilized for optimizing the ANN

| Parameters | Values |
| --- | --- |
| Population size | 80 |
| Maximum number of iterations | 150 |
| Search range for weights and biases | [-3.0,3.0] |

## 4. Definitions of model evaluation metrics

$$R^2 = \frac{\sum_{i=1}^{n}(\hat{y}_i - \bar{y})^2}{\sum_{i=1}^{n}(y_i - \bar{y})^2} \tag{S8}$$

$$\text{ARE} = \frac{|y_i - \hat{y}_i|}{y_i} \times 100\% \tag{S9}$$

$$\text{MARE} = \frac{100\%}{n} \times \sum_{i=1}^{n} \frac{|y_i - \hat{y}_i|}{y_i} \tag{S10}$$

Where $y_i$ is the experimental value, $\hat{y}_i$ is the predicted value, $\bar{y}$ is the mean of the experimental values, and $n$ is the number of data points.

## 5. Line-width consistency and repeatability in curved-surface printing

To evaluate the consistency and repeatability of the line width during curved-surface printing, three repeated printing trials are conducted for each target line width of 30, 60, and 90μm. In each trial, five measurement points are selected within each of the starting, middle, and end segments of the printing path. **Figure S2** presents the measured line widths at these representative positions, while **Table S3** summarizes the statistical results obtained from the three repeated printing trials. The overall coefficients of variation for the target line widths of 30, 60, and 90μm are 4.54%, 3.69%, and 1.41%, respectively. For the three target line widths, the maximum differences among the mean line widths at the starting, middle, and end segments are 0.94, 1.39, and 1.32μm, respectively, while the maximum differences among the overall mean line widths obtained from the three repeated printing trials are 0.08, 1.12, and 1.29μm, respectively. These results indicate limited line-width variation among different positions on the curved surface and close agreement among the repeated printing trials, demonstrating the good positional consistency and printing repeatability of the proposed method.

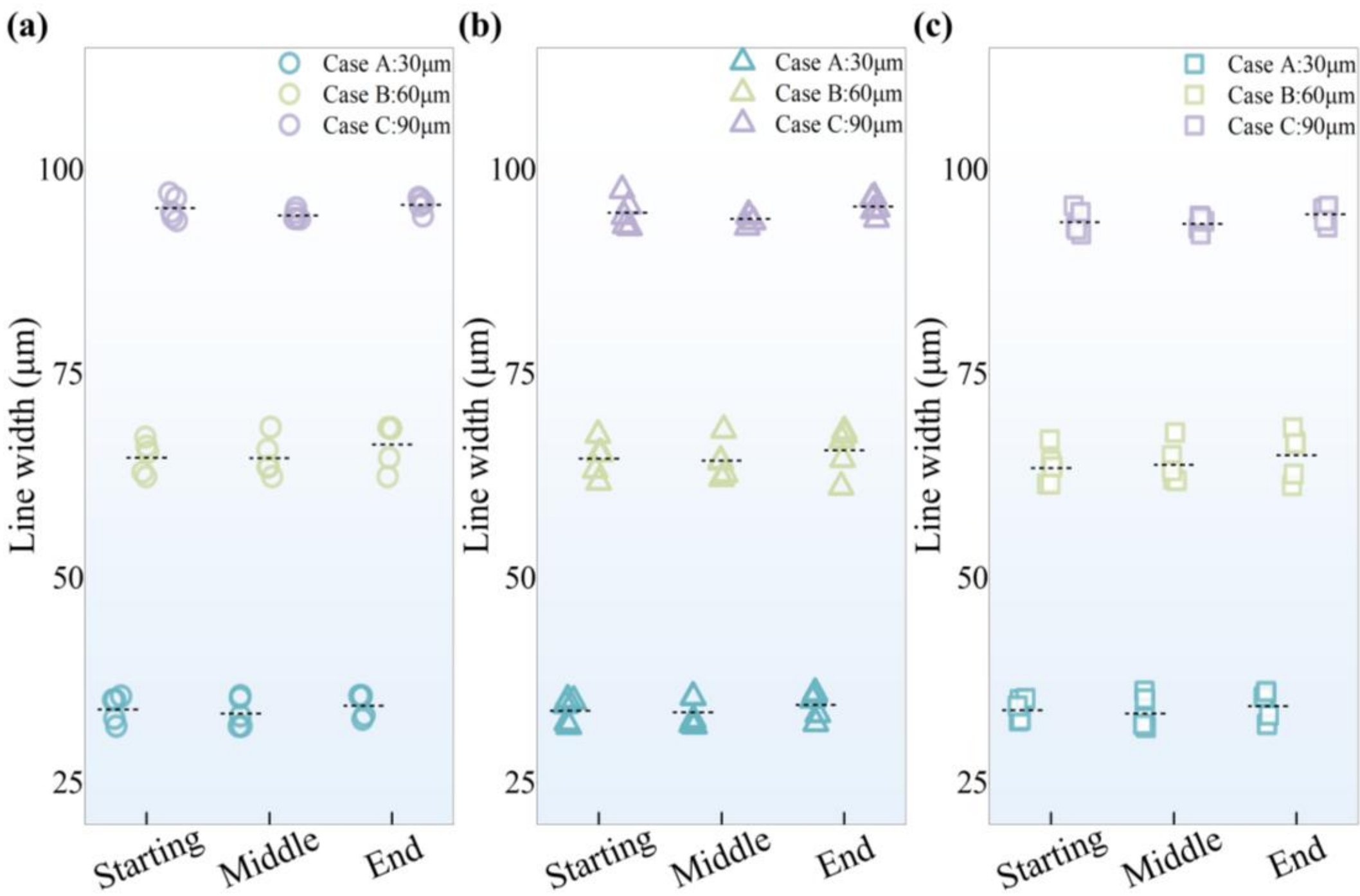


Figure S2 Line-width measurements. (a) first printing; (b) second printing; (c) third printing. (the short dashed lines indicate the corresponding mean values)

Table S3 Overall variability of the measured line widths

| Target line width (μm) | Standard deviation (μm) | Coefficient of variation (%) |
|---|---|---|
| 30 | 1.55 | 4.54 |
| 60 | 2.39 | 3.69 |
| 90 | 1.33 | 1.41 |

## 6. Improvement of printing stability and deposited-line morphology using the ML-AE-EHD printing method

To further visually evaluate the improvement in curved-surface printing quality achieved by the ML-AE-EHD printing method, **Figure S3** compares the printing processes and deposited-line morphologies obtained without regulation and with ML-AE-EHD printing method regulation. **Figure S3(a, b)** shows the liquid-bridge states at different positions along the printing path before and after regulation. Without regulation, the liquid bridge exhibits varying degrees of deflection as the printing position changes. In contrast, with ML-AE-EHD printing method regulation, no obvious liquid-bridge deflection is observed at the different positions, indicating improved printing stability. **Figure S3(c, d)** further compares the corresponding deposited-line morphologies. Compared with the unregulated results, the regulated lines exhibit more uniform widths and better overall morphological consistency. These image comparisons demonstrate that ML-AE-EHD printing effectively improves both the stability of the curved-surface printing process and the consistency of the deposited lines.

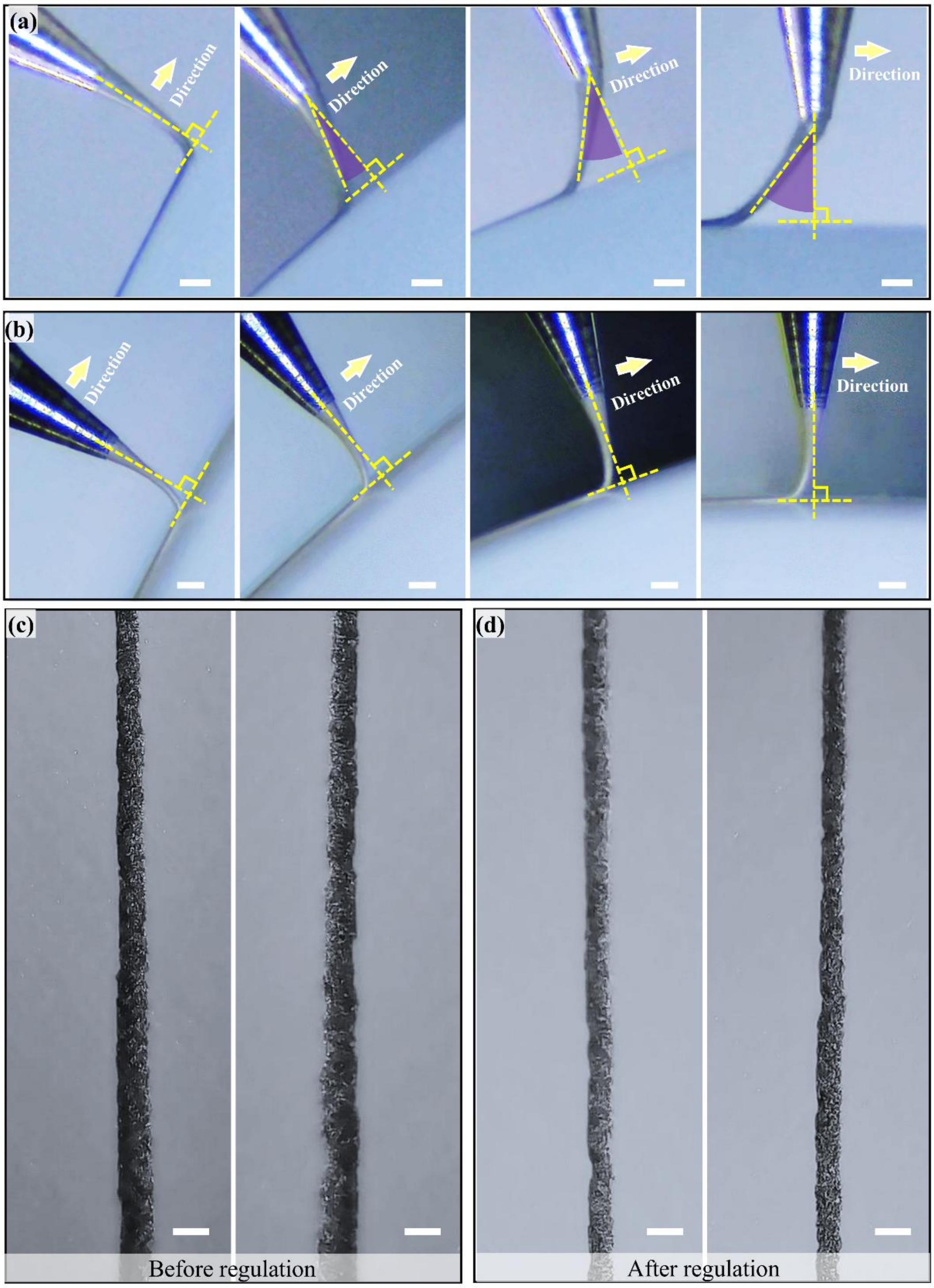


Figure S3 Comparison of curved-surface printing stability and deposited-line morphology. (a) liquid-bridge states without regulation; (b) liquid-bridge states with regulation; (c) deposited line morphologies without regulation; (d) deposited line morphologies with regulation. Scale bars, 100μm.

## 7. Geometric dimensions of the curved FSS and antenna

Table S4 Dimensions of the curved FSS.

| Dimension | Values(mm) |
|---|---|
| *R1* | 150 |
| *Φ1* | 143 |
| *Φ2* | 5.85 |
| *Φ3* | 6.15 |

Table S5 Dimensions of the curved antenna.

| Dimension | Values(mm) |
|---|---|
| *H* | 50.0 |
| *L* | 69.5 |
| *R2* | 50.0 |
| *d1* | 48.5 |
| *d2* | 18.5 |
| *d3* | 29.1 |
| *l1* | 23.5 |
| *l2* | 50.0 |
| *r* | 10.0 |
| *Φ4* | 13.5 |
| *Φ5* | 8.0 |

## 8. Line-width accuracy and consistency of the printed conformal FSS

To further evaluate the dimensional accuracy and consistency of the printed conformal FSS, line-width measurements are conducted at 100 locations randomly selected throughout the printed resonant microstructure array. As shown in **Figure S4**, the measured line widths are primarily distributed around the value of 150 μm. The mean line width and standard deviation are 150.09 μm and 3.47 μm, respectively, and the mean absolute error relative to the designed line width is 2.65 μm, corresponding to a mean absolute relative error of 1.76%. Among the 100 measurement points, 96% of the measured line widths fall within the range of 145-155 μm. These results

demonstrate that the printed resonant microstructure array exhibits good line-width accuracy and dimensional consistency.

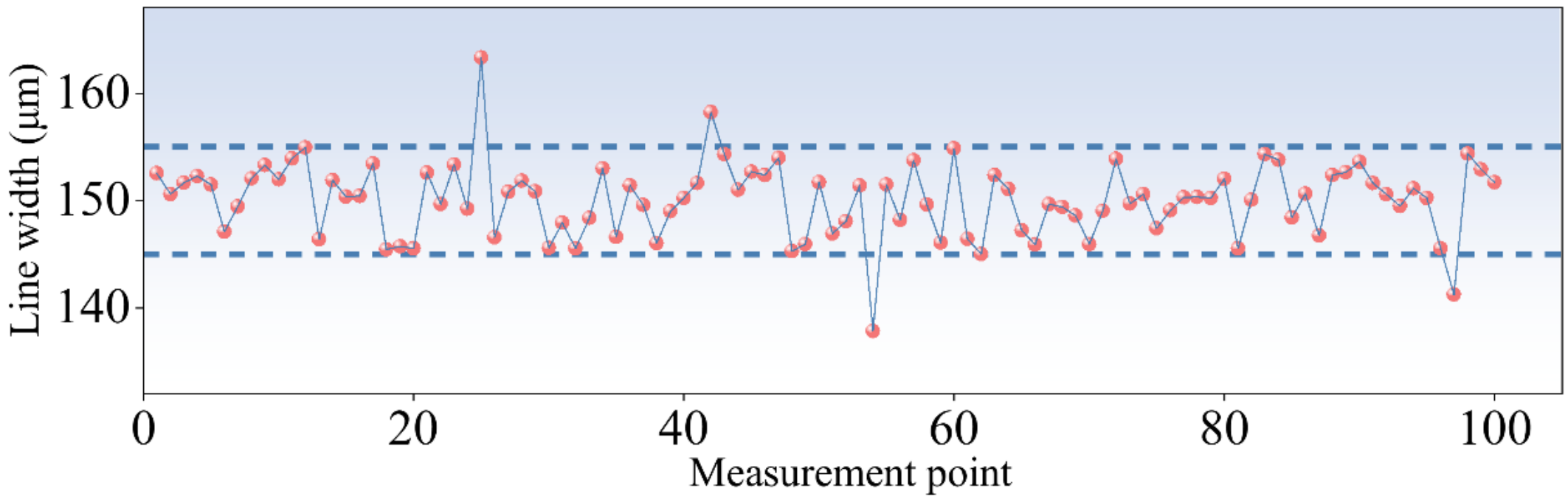


Figure S4 Measured line width of the printed conformal FSS.
